# Subtype C HIV-1 reservoirs throughout the body in ART-suppressed individuals

**DOI:** 10.1172/jci.insight.162604

**Published:** 2022-10-24

**Authors:** Zhou Liu, Peter Julius, Guobin Kang, John T. West, Charles Wood

**Affiliations:** 1 School of Biological Sciences, University of Nebraska-Lincoln, Lincoln, Nebraska, USA.; 2 Department of Interdisciplinary Oncology, Louisiana State University Health Sciences Center, Louisiana Cancer Research Center, New Orleans, Louisiana, USA.; 3 Department of Pathology and Microbiology, School of Medicine, University of Zambia, Lusaka, Zambia.

**Keywords:** AIDS/HIV, Virology, AIDS vaccine, Molecular biology

## Abstract

Subtype B HIV-1 reservoirs have been intensively investigated, but reservoirs in other subtypes and how they respond to antiretroviral therapy (ART) is substantially less established. To characterize subtype C HIV-1 reservoirs, we implemented postmortem frozen, as well as formalin fixed paraffin embedded (FFPE) tissue sampling of central nervous system (CNS) and peripheral tissues. HIV-1 *LTR*, *gag*, *envelope* (*env*) DNA and RNA was quantified using genomic DNA and RNA extracted from frozen tissues. RNAscope was used to localize subtype C HIV-1 DNA and RNA in FFPE tissue. Despite uniform viral load suppression in our cohort, PCR results showed that subtype C HIV-1 proviral copies vary both in magnitude and tissue distribution, with detection primarily in secondary lymphoid tissues. Interestingly, the appendix harbored proviruses in all subjects. Unlike subtype B, subtype C provirus was rarely detectable in the CNS, and there was no detectable HIV-1 RNA. HIV-1 RNA was detected in peripheral lymphoid tissues of 6 out of 8 ART-suppressed cases. In addition to active HIV-1 expression in lymphoid tissues, RNAscope revealed HIV RNA detection in CD4-expressing cells in the appendix, suggesting that this tissue was a previously unreported potential treatment-resistant reservoir for subtype C HIV-1.

## Introduction

HIV can infect CD4-expressing immune cells and eventually cause AIDS. AIDS was universally fatal with opportunistic coinfections due to the loss of CD4 T cell–mediated immunity and HIV-associated neurocognitive disorder (HAND), which includes severe HIV-associated dementia (HAD) (1, 2). Although the introduction of combined antiretroviral therapy (cART) has turned that once fatal disease into a managed care scenario, it is not a cure; it is accompanied with some side effects, and life-long treatment is required (3). There are 2 HIV types, HIV-1 and HIV-2, with HIV-1 accounting for more than 95% of all worldwide HIV infections (4). Phylogenetics segregates HIV-1 into 4 groups: M (major group), O (outlier group), N (non-M, non-O), and P (rare subtype pending confirmation). Group M HIV-1 is responsible for more than 90% of all HIV infections and is subdivided in 10 separate subtypes: A, B, C, D, F, G, H, J, and K, as well as circulating recombinant forms (CRFs) that are essentially hybrid subtypes formed by recombination between at least 2 subtypes (5). These subtypes are unevenly distributed throughout the world, with subtype C being the most widespread. Subtype B HIV-1 is the predominant subtype in Europe, North America, Japan and Australia, but it accounts for only 12% of global HIV infections. In contrast, subtype C HIV-1 is prevalent in sub-Saharan Africa and India and is responsible for more than 50% of all HIV infections (6). Investigations of HIV latency in non–subtype B lineages has been limited and is now important, as effective treatment is increasingly available and utilized in areas where non–subtype B genotypes predominate.

Despite the fact that cART can suppress HIV-1 plasma viral load (pVL) to an undetectable level (<50 copies/mL) in a majority of treated people living with HIV (PLWH), it cannot eliminate HIV-1 from the body (7). This is because HIV reverse transcribes its RNA genome into a complimentary double-stranded DNA that then integrates into the host genome as a provirus, and it is the presence of this potentially replicable provirus that constitutes the latent HIV tissue reservoir. Establishment of HIV-1 reservoirs is thought to occur early after infection in different locations throughout the body (7). HIV-1 proviruses in different reservoirs might stay latent or may be persistently expressed, which means a viral reservoir could be: (a) an active persistently expressing reservoir, (b) a silent but intact proviral reservoir, or (c) a reservoir composed of defective integrated provirus that may or may not have the capacity to express HIV-1 genes (8). However, it would seem likely that real tissues reservoirs could potentially contain all 3 “states” of HIV-1 persistence. In some HIV-1 tissue reservoirs, it is difficult to achieve a high penetrance of ART due to drug chemistry and physiological barriers. One example is the blood-brain barrier (BBB), which can limit some ART drugs’ access to the CNS (9). In addition, poor ART penetrance could result in localized persistent low-level replication in some tissues (persistent reservoir). Recent studies suggest that proviral DNA levels in PBMC decline with a half-life estimated around 12 years, and they are not differentially affected by the specific antiretroviral regimen used to achieve and maintain viral suppression (10–12). However, many studies have demonstrated that HIV will rebound after cessation of ART, and the rebounding virus originates from persistent reservoirs or reactivated latent reservoirs (13, 14). Therefore, lifelong ART is necessary, which carries both a physiological and economic burden, especially in low-resource areas, such as sub-Saharan Africa.

Due to demonstration of a viral reservoir in PBMC and the difficulty in sampling other potential reservoir tissues, most HIV latency research has focused on large volume blood samples and lymphoid tissues obtained by biopsy from subtype B HIV-1–infected individuals (13, 15, 16), while some other studies alternatively utilized a nonhuman primate (NHP) model or a humanized mice model (17–21). Those studies have suggested that CNS tissues (17, 22–26), bone marrow (BM) (27–30), lung (31, 32), kidney (33), pancreas (34), prostate (35, 36), gastrointestinal (GI) tract tissue (37–39), reproductive tract tissue (40–42), and adipose tissue (43) serve as subtype B HIV-1 tissue reservoirs. However, as far as we know, only 2 studies had investigated HIV tissue reservoirs that utilized tissues from different anatomical locations of the same individual, including lymphoid, brain, and GI tract tissues, and both were on subtype B HIV-1 (34, 44).

The challenges of collecting high-quality systematic postmortem human specimens from endemic areas to support molecular investigations had previously limited efforts to define subtype C HIV-1 reservoirs. It has been reported that subtype C HIV-1 can be detected at high levels in peripheral blood mononuclear cells (PBMCs) of ART naive individuals (45). Our laboratory has also found that subtype C HIV-1 proviral DNA can be detected at low and variable levels in CNS tissues, regardless of ART success or failure (46), but so far, there has been little systematic characterization of the distribution of this subtype among major human organ systems, especially in those who are ART-experienced with pVL suppression. Whether the magnitude, distribution, or state of activation of subtype C HIV-1 reservoirs is similar or dissimilar to that in HIV subtype B is unknown.

In this study, various flash-frozen and formalin fixed paraffin embedded (FFPE) brain and peripheral tissues were collected postmortem to support quantification of HIV reservoirs in 8 subtype C HIV-1–infected aviremic Zambian individuals. Our results reveal that there are very few tissues harboring subtype C HIV-1 proviral DNA and potential intact viral genome in the brain, and all are at a low copy number. Moreover, there is no detectable viral RNA in the brain, indicating that, in contrast to subtype B, the brain is not a robust reservoir for HIV-1 subtype C. In the peripheral tissues, the HIV-1 subtype C proviral reservoir distribution and magnitude varied among different tissues and individuals with lymphoid tissues harboring consistent viral burden in most individuals, as anticipated. Notably, we detected a robust HIV-1 tissue reservoir in the appendix of 100% of individuals, which is, to our knowledge, the first report of this tissue as a site of HIV persistence in treated individuals. Lymph nodes (LNs) were the major compartments where persistent viral RNA expression was detected, suggesting that ART fails to eliminate HIV-1 production from these tissues, despite pVL suppression. Furthermore, using RNAscope, viral DNA and RNA can be detected in parenchymal appendix tissue, where it is colocalized with CD4-expressing cells. Evaluation of subtype C HIV-1 reservoirs in this study addresses crucial gaps in our understanding of the magnitude and unique distribution of subtype C HIV-1 tissue reservoirs in the context of viral suppression. Such understanding may contribute to a greater capacity to understand HIV-1 infection and to create better future strategies for HIV cure.

## Results

### Tissue samples and subtype determination in ART-suppressed aviremic individuals.

**Figure 1:**
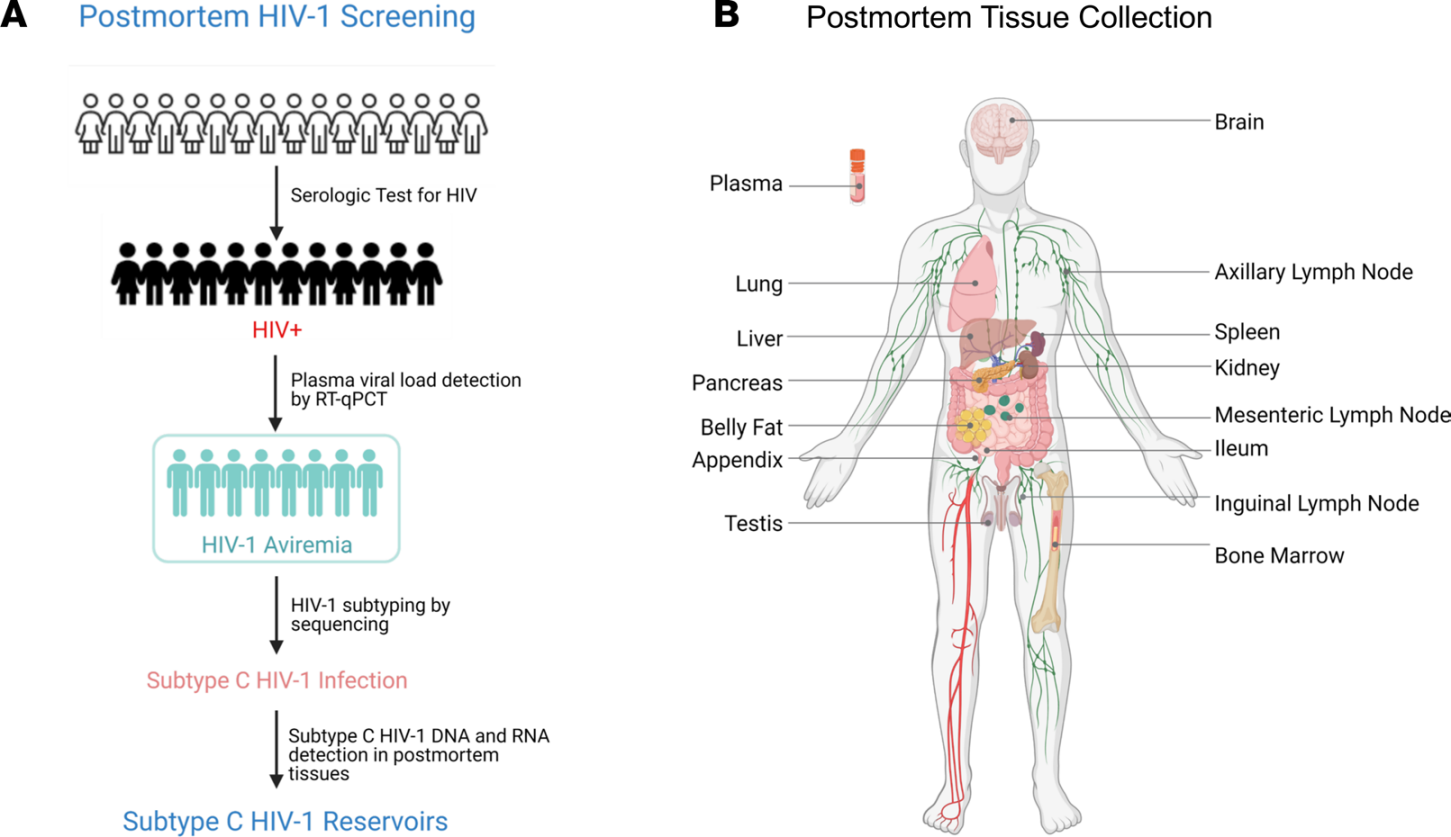
HIV-1 aviremic cohort and postmortem sample collection. (**A**) The flow diagram showed postmortem HIV-1 screening. HIV serologic tests were conducted on plasma samples of all postmortem cases to identify HIV-1–infected individuals. qPCR was utilized for plasma viral load detection, and those cases with undetectable plasma viral load were then subjected for HIV-1 subtyping to confirm clade C HIV-1 infection in those aviremic individuals. Eventually, an 8-case cohort was identified to be infected with subtype C HIV-1. Viral DNA and RNA copies were determined by different PCR-based methods from frozen tissue genomic DNA and total RNA. To characterize more detail for subtype C HIV-1 reservoirs, RNAscope was performed on representative FFPE tissues. (**B**) Postmortem samples were collected within 48 hours of a subject’s death. Half of each brain and peripheral tissue was collected and processed into a frozen sample, while the other half was processed into a FFPE sample. Frozen plasma samples were also collected.

The experimental design highlighting the collected postmortem sampling in this study is shown in Figure 1A. Serologic testing for HIV was conducted for screening HIV-1–infected individuals using femoral or cardiac plasma samples. Plasma RNA preparations from HIV-1^+^ cases were subjected to standard quantitative PCR (qPCR) to quantify pVLs. Eight male individuals were identified as seropositive but lacking detectable pVLs (< 70 copies/mL) and were defined as “aviremic” (Table 1). The samples listed in Figure 1B, including 8 CNS tissues (frontal lobe, parietal lobe, temporal lobe, occipital lobe, hippocampus, cerebellum, basal ganglia, and choroid plexus [CPx]) and 13 peripheral tissues (mesenteric LN, axillary LN, inguinal LN, spleen, BM, ileum, appendix, liver, kidney, lung, pancreas, belly fat, testis), and plasma were collected within 48 hours of the aviremic individual’s death. The median age of the 8 patients at death was 46 (range from 39 to 60). Among the 8 aviremic individuals, information about ART duration was obtained for 6 individuals (information missing for cases 3 and 7) whose median duration of ART was 6.5 years (range from 4 to 10 years). Self-reported data indicate that, after being commenced on ART, those 6 individuals received ART until their deaths (Table 1). In Zambia, Atripla — which is a combination of efavirenz, emtricitabine, and tenofovir disoproxil fumarate in 1 tablet form — is the only drug being given for uniform ART in government clinics and served as the first line of treatment that all the decedents in our cohort received. Tissues from all 8 aviremic, presumably ART-suppressed, individuals were evaluated for HIV reservoirs.

To first determine the HIV-1 subtype and to validate HIV serologic testing on the 8 aviremic cases, we subjected equivalent genomic DNA extracts from frozen postmortem tissues to nested PCR for the HIV-1 *env* gene. The HIV-1 *env* gene sequences were amplified from at least 1 peripheral tissues for all individuals, and those amplification products were gel purified and then sequenced using primers against the HIV-1 gp120 V3 loop. Application of recombinant identification program (RIP) and a BLAST-based HIV-1 genotyping tool to *env* sequence alignments revealed the highest alignment with HIV-1 subtype C (Table 1 and Supplemental Table 1; supplemental material available online with this article; https://doi.org/10.1172/jci.insight.162604DS1).

### The brain is not a good reservoir for subtype C HIV-1 in aviremic individuals.

After subtyping, we proceeded to identify tissues harboring subtype C HIV-1 provirus. Genomic DNA samples from frozen postmortem tissues were tested for quality through cellular gene *GAPDH* amplification and were then subjected to qPCR with primers against the HIV-1 *LTR*, *gag*, and *env* DNA. Similar amplification efficiencies of those 3 pairs of primers had been validated through qPCR utilizing 8E5 genomic DNA, which carries a single copy of integrated HIV genome (Supplemental Table 2).

**Figure 2:**
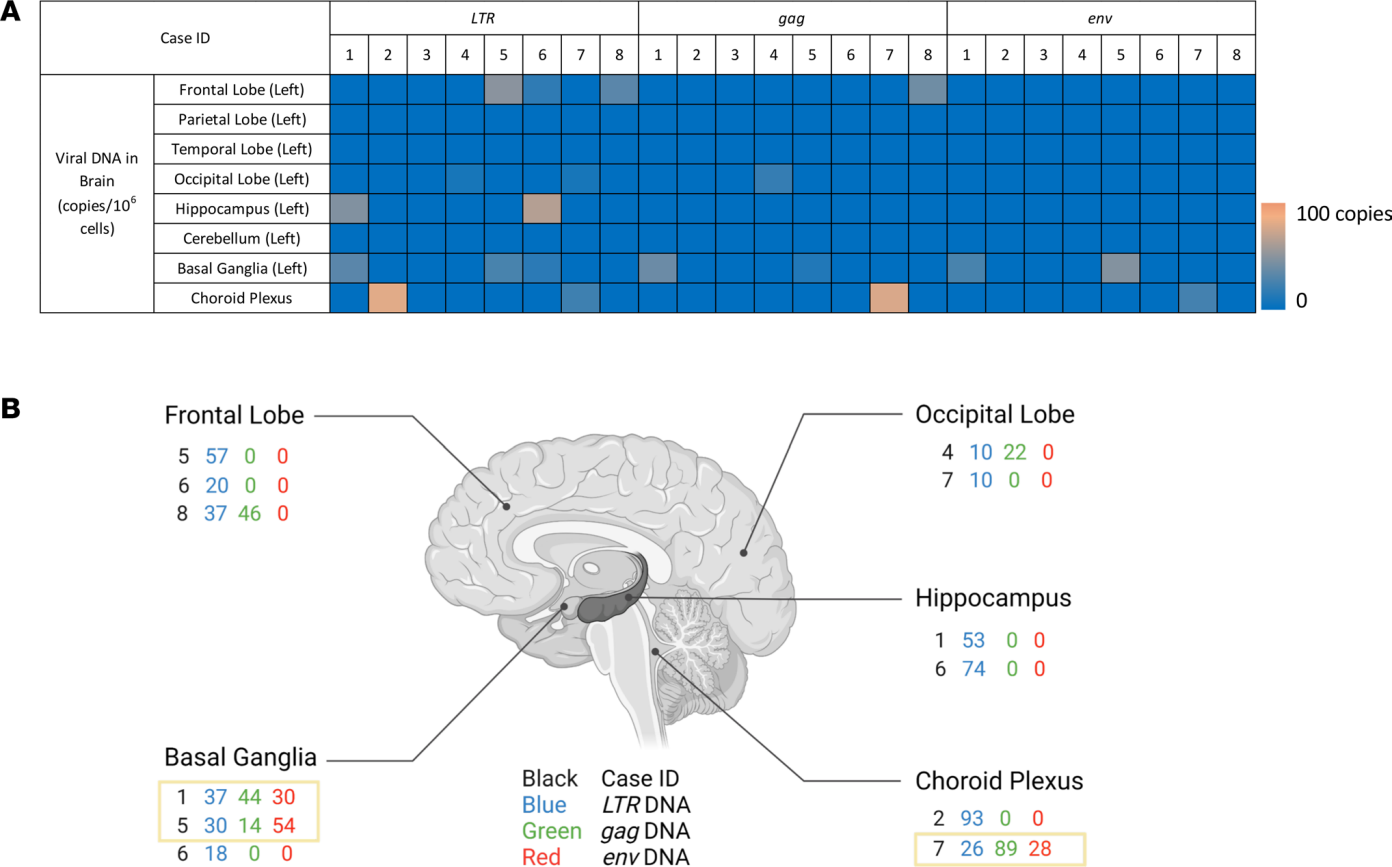
Subtype C HIV-1 DNA in aviremic brain tissues. (**A**) Heatmap of subtype C HIV-1 DNA in aviremic brain tissues. A heatmap displaying the abundance of 3 subtype C HIV-1 DNA (*LTR*, *gag*, and *env*) in different brain regions of 8 aviremic subjects was generated by qPCR analysis. Blocks with colors indicate undetectable (0) DNA copy (blue) to increase DNA copies (brown, up to 93 copies/1 × 10^6^ cells). CNS tissues from the same hemisphere were analyzed (left). (**B**) Copy numbers of subtype C HIV-1 DNA detected in aviremic brain. The different regions harboring viral DNA and the viral DNA copy numbers in the brain of the 8 aviremic subjects were shown. There was no detectable viral DNA in the parietal lobe, temporal lobe, and cerebellum of 8 aviremic cases. HIV-1 DNA copies were identified by qPCR with 100 ng genomic DNA as a template. HIV-1 *LTR* (blue), *gag* (green), and *env* (red) DNA copy numbers were calculated as the mean of triplicate qPCR reactions and normalized to 1 million cells. The rectangles mark 3 tissues that have all 3 viral genes, indicating potential intact viral genome in those tissues.

The brain has been documented as a reservoir for subtype B HIV-1, but it is unclear whether it is a robust reservoir for other subtypes, including subtype C. Our qPCR results show that, among 64 tested brain tissue samples (8 tissues for each individual), subtype C HIV-1 *LTR* DNA was only detected in 12 (18.8%) brain tissue samples. The frontal lobe and basal ganglia are the 2 CNS sites with highest frequency in harboring HIV-1 proviruses. However, the HIV-1 viral DNA copies in the brain vary in different individuals and different brain regions (Figure 2 and Supplemental Figure 1). Particularly, subtype C HIV-1 *LTR* DNA was detected in the frontal lobe of cases 5, 6, and 8 with 57, 20, and 37 viral DNA copies/1 × 10^6^ cells, respectively; it was detected in the basal ganglia of cases 1, 5, and 6 with 37, 30, and 18 viral DNA copies/1 × 10^6^ cells, respectively. Furthermore, subtype C HIV-1 *LTR* DNA were also detected in the occipital lobe of cases 4 and 7, the hippocampus of cases 1 and 6, and the CPx of cases 2 and 7, whereas there was no detectable viral *LTR* DNA in the parietal lobe, temporal lobe, or cerebellum. Among those 12 HIV-1 *LTR* DNA harboring brain tissue samples, case 8 frontal lobe, cases 1 and 5 basal ganglia tissues, case 4 occipital lobe, and case 7 CPx were identified to contain HIV-1 *gag* DNA with 46, 44, 14, 22, and 89 viral DNA copies/1 × 10^6^ cells, respectively. Eventually, besides harboring HIV-1 *LTR* and *gag* DNA, cases 1 and 5 basal ganglia tissues and case 7 CPx were found to harbor HIV-1 *env* DNA with 30, 54, and 28 viral DNA copies/1 × 10^6^ cells, respectively (Figure 2B). The presence of all 3 subtype C HIV-1 genes implied the potential full-length or nearly full-length subtype C HIV-1 proviruses in those 3 brain tissue samples, while the presence of viral *LTR* DNA but lack of *gag* or *env* DNA indicated probable existence of defective HIV-1 reservoirs in the other 9 of 12 viral DNA–harboring brain tissue samples (75%).

Besides showing a variation among different CNS tissues, the frequencies and the copies of subtype C HIV-1 proviruses in the CNS also varied among different individuals. In general, subtype C HIV-1 *LTR* DNA was detected in at least 1 brain tissue in 7 of 8 cases (87.5%), while viral provirus was not detectable in any brain tissue from case 3. Three brain regions from case 6, the frontal lobe, hippocampus, and basal ganglia were identified as tissues harboring subtype C HIV-1 provirus. Moreover, each case 2, 4, and 8 has only 1 brain tissue sample (the CPx, occipital lobe, and frontal lobe, respectively) that contained HIV-1 provirus. However, cases 1, 5, and 7 have 2 different brain tissue samples with detectable HIV-1 proviruses (Figure 2A and Supplemental Figure 1). Among those 7 individuals harboring viral *LTR* DNA in brain, 5 cases (1, 4, 5, 7 and 8) harboring viral *gag* DNA were identified, and 3 cases (1, 5, and 7) were further determined to contain viral *env* DNA in the brain. Notably, the viral DNA copies in brain tissue samples were all lower than 100 copies/1 × 10^6^ cells after normalized to 1 million cells (Figure 2B and Supplemental Figure 1). The medians of detectable viral DNA copies were all 0, no matter whether they were in the same brain region of different individuals or in different brain regions of same individual. Moreover, we did not observe a specific pattern regarding the distribution of subtype C HIV-1 reservoirs in the brain.

Despite the rare and low subtype C HIV-1 viral DNA detected in aviremic brain, we wanted to know whether those proviruses express any viral RNA. However, according to the results of 1-step reverse transcription (RT)-qPCR utilizing total RNA that extracted from brain tissue samples harboring viral DNA and RNA sample quality confirmed by cellular RNA peptidyl-prolyl cis-trans isomerase B (*PPIB*) amplification, there was no detectable viral RNA (*LTR*, or *gag,* or *env* RNA) in any aviremic brain region (Supplemental Figure 2).

### Detection of subtype C HIV-1 peripheral tissue reservoirs.

**Figure 3:**
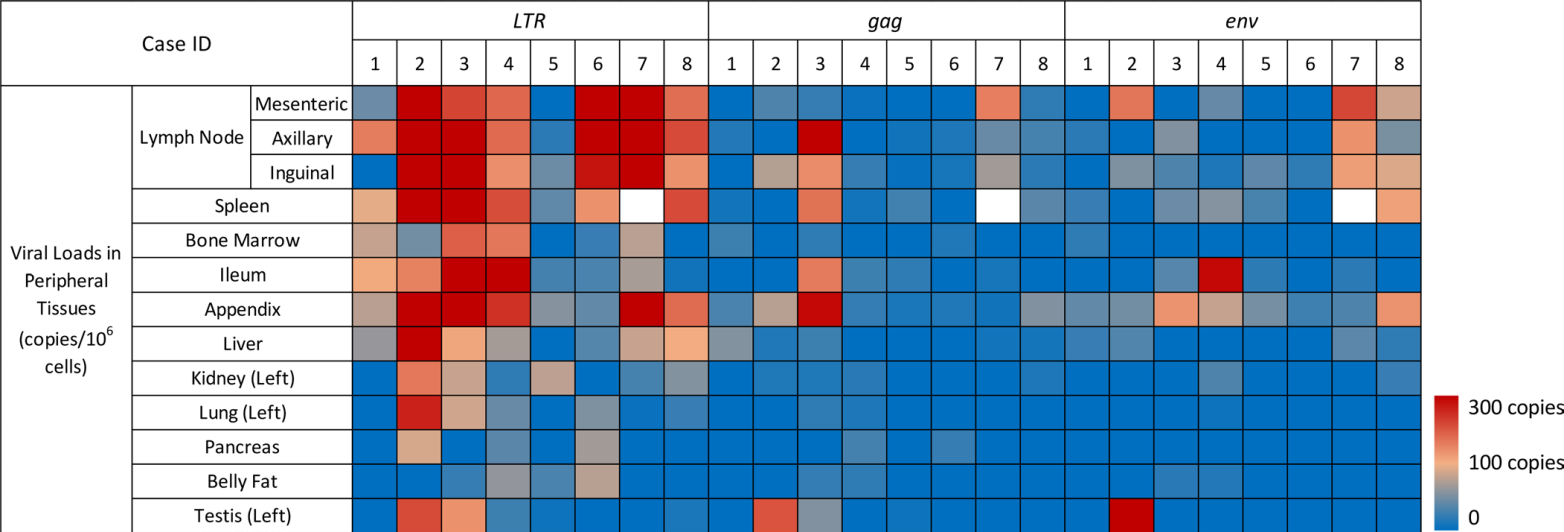
Heatmap of subtype C HIV-1 DNA in aviremic periphery. A heatmap displays the abundance of 3 subtype C HIV-1 DNA (*LTR*, *gag*, and *env*) in peripheral anatomical locations of the 8 aviremic subjects based on qPCR analysis. Blocks with colors indicate undetectable (0) DNA copy (blue) to increased DNA copies (up to 1538 copies/1 × 10^6^ cells). Spleen of case 7 was failed to be sampled (blank cell). HIV-1 DNA copies were identified by qPCR with 100 ng genomic DNA as a template. All samples were analyzed in triplicate qPCR reactions, and viral DNA copy numbers were calculated as the mean of triplicate and normalized to 1 million cells.

In contrast to the rare presence in the brain, subtype C HIV-1 proviruses were extensively detected in different peripheral tissues throughout the body, and the overall copies of viral DNA detected in peripherial tissue was remarkably higher than that in the brain (*P* < 0.001) (Figure 2A, Figure 3, Table 2, and Supplemental Table 3A). Moreover, 82 of 103 peripheral tissues (79.6%) were identified harboring HIV-1 DNA, and this frequency is also significantly higher than that in the brain (18.8%) (*P* < 0.0001) (Figure 3 and Supplemental Table 3B). Unlike the brain, no specific peripheral tissue was found to be completely free of viral DNA. Furthermore, there is no correlation between the viral burden in brain and peripherial tissue.

Subtype C HIV-1 proviral DNA copies varied in different peripheral tissues, indicating different proviral reservoir sizes. Notably, of the 13 types of peripheral tissues analyzed, only 2 types of lymphoid tissue, the LNs and spleen, and 2 types of GI tract tissue, the ileum and appendix, were found to contain viral *LTR* DNA in all aviremic individuals (Figure 3 and Supplemental Figure 3). Furthermore, compared with other peripheral tissues, LNs, spleen, ileum, and appendix showed higher viral *LTR* DNA burdens, with the median of *LTR* DNA copies at 308, 213, 88, and 211 copies/1 × 10^6^ cells, respectively (Table 2 and Supplemental Table 4). Another lymphoid tissue, BM, was found to have viral *LTR* DNA in 6 aviremic individuals with the median *LTR* DNA copies of 62 copies/1 × 10^6^ cells. Other tissues involve in different human organ systems were also found to harbor HIV-1 *LTR* DNA. They include the liver, kidney, lung, pancreas, adipose tissue, and testis, which have the median of HIV-1 *LTR* DNA copies at 75, 41, 33, 0, 11, and 8 copies/1 × 10^6^ cells, respectively. The highest viral *LTR* DNA copy is 1,538 copies/1 × 10^6^ cells in the mesenteric LN of case 7 (Table 2). Among the 82 HIV-1 *LTR* DNA harboring peripheral tissues, 56 tissues were found to also contain HIV-1 *gag* DNA, and 43 tissues were further identified to harbor HIV-1 *env* DNA, indicating that intact HIV-1 provirus might be integrated into the host genome of those 43 tissues. Interestingly, only the LNs and appendix were determined to harbor all 3 proviral genes in all aviremic cases (Table 2 and Supplemental Figure 3). Proviruses that harbor HIV-1 *LTR*, *gag*, and *env* DNA were also found in the spleen of cases 1, 3, 4, 5, and 8. In addition, potential intact proviruses were also detected in the bone marrow, ileum, liver, kidney, lung, adipose tissue, and testis of some cases, whereas there was no detectable viral *env* DNA in any pancreas tissue (Table 2 and Supplemental Figure 3).

**Figure 4:**
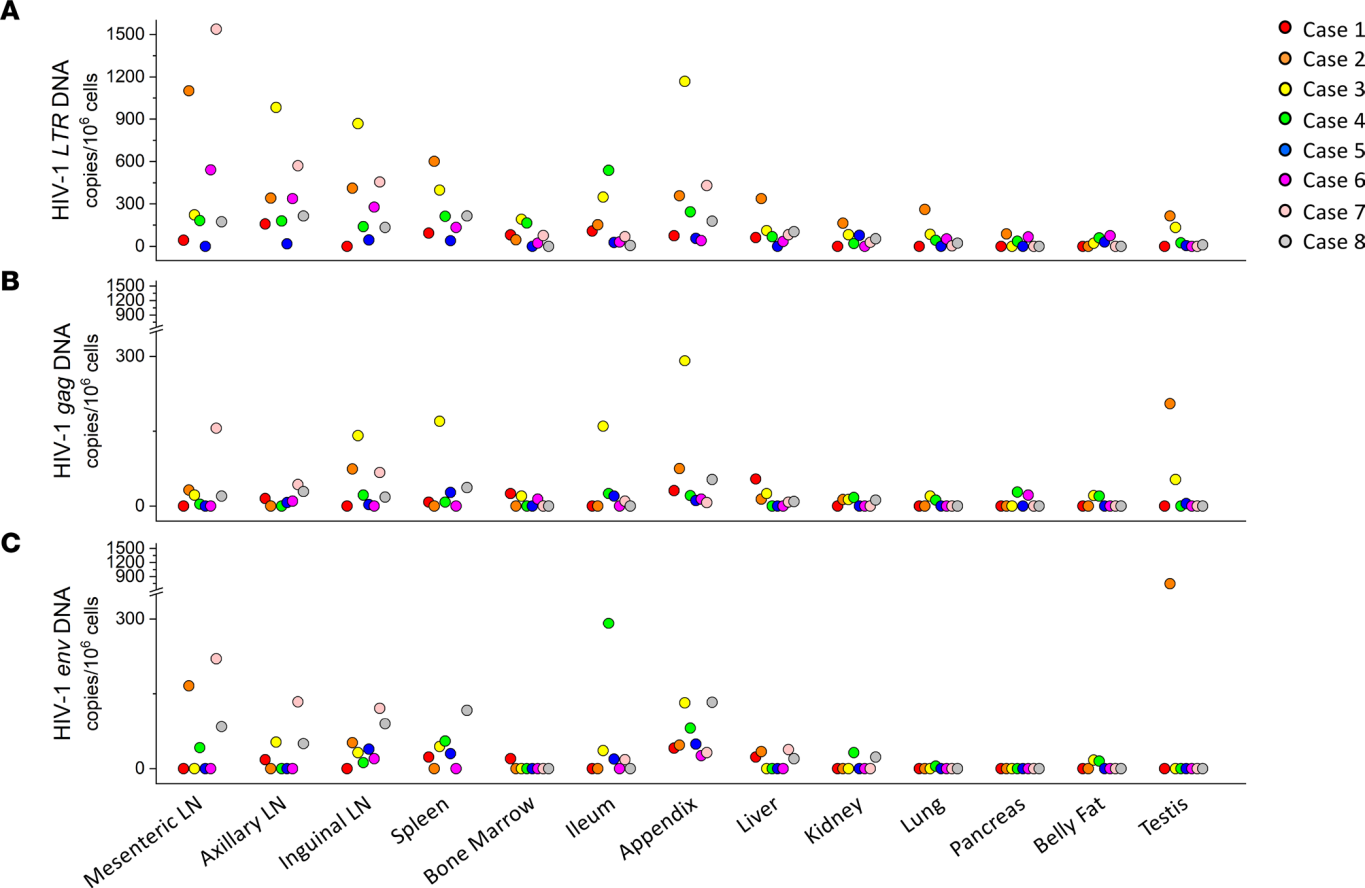
Subtype C HIV-1 DNA quantification in peripheral anatomical locations. Scatter plots sorted by different peripheral tissues and colored for different subjects. The *y* axis showed HIV-1 DNA copies/1 × 10^6^ cells. (**A**) HIV-1 *LTR* DNA in the periphery. (**B**) HIV-1 *gag* DNA in the periphery. (**C**) HIV-1 *env* DNA in the periphery. In total, 100 ng genomic DNA was used in each qPCR reaction as template. The dots represent the means of triplicate qPCR reactions for the HIV-1 DNA copies.

The comparisons of the variations in the proviral DNA copy number among different tissues from different cases are shown in Figure 4. In scatter plots, quantified viral DNA copy numbers from tissues of cases 1–8 were indicated as red, orange, yellow, green, blue, purple, pink, and gray dots, respectively. In general, 3 cases with relatively shorter postmortem interval (PMI) — cases 2 (7 hours), 3 (3 hours), and 4 (4 hours) — had relatively higher overall viral *LTR* DNA burden than that in 3 other cases with longer PMI — cases 1 (30 hours), 6 (26 hours), and 8 (33 hours) (Figure 4A and Supplemental Figure 4). Moreover, although the PCR detection may have limited our ability to capture extremely low levels of viral DNA in some tissues, we have detected more tissues harboring HIV-1 *LTR* DNA for cases 2, 3, and 4 than cases 1, 6, and 8 (Figure 4A). Particularly, viral *LTR* DNA was detected in nearly every peripheral tissue of cases 2, 3, and 4, except the belly fat of case 2 and pancreas of case 3 (Figure 4A and Table 2). In contrast, case 1 had the least peripheral tissues with detectable HIV-1 DNA. However, except for displaying a negative correlation in ileum (correlation coefficient = –0.8015, *R*^2^ = 0.6424), we did not observe the correlation of PMI with levels of viral DNA detected in other peripheral tissues (Supplemental Figure 5). With regard to potential intact proviruses, cases 4 and 8 have most peripheral tissues — 8 and 7 tissues, respectively — harboring HIV-1 *LTR*, *gag*, and *env* DNA, whereas case 6 only had 2 peripheral tissues (inguinal LN and appendix) containing all 3 viral genes. Additionally, case 5 displayed lowest overall subtype C HIV-1 reservoir magnitude possessing < 100 copies/1 × 10^6^ cells of proviral DNA in every HIV-1 provirus containing peripheral tissues (Figure 4 and Table 2). For most cases, those with higher viral DNA copies in the LNs appeared to have higher viral DNA copies in other peripheral tissues, except for cases 6 and 7, whose viral DNA copies in LN were high but low in other peripheral tissues compared with other cases.

To confirm our qPCR results, we also performed ultrasensitive digital PCR (dPCR) to obtain absolute HIV-1 DNA copies. Considering the similar amplification efficiencies of *LTR*, *gag*, and *env* primers and the higher overall viral *LTR* DNA copies in cases 2, 3, and 4, we have utilized the same *LTR* primers and the same batch of genomic DNA used in qPCR from the occipital lobe, mesenteric LN, appendix, and testis of cases 2, 3, and 4 to perform dPCR. The dPCR results showed good correlation with qPCR results for both CNS tissue occipital lobe and 3 other peripheral tissues (Supplemental Figure 6 and Supplemental Table 5). Overall, it showed a positive qPCR/dPCR efficiency correlation with a 0.7054 *R*^2^ value (Supplemental Figure 6B). The dPCR results further confirmed and strengthened our findings by qPCR.

### Persistent expression of subtype C HIV-1 proviruses in the peripherial tissues.

It is well documented that defective integration is common in retroviruses (47–50); the presence of viral *LTR* DNA but lack of *gag* or *env* DNA in some tissues of our cohort suggests the existence of defective subtype C HIV-1 reservoirs in those tissues, while the higher *LTR* DNA copies compared with *gag* or *env* DNA copies in most tissues suggests that intact and defective subtype C HIV-1 proviruses might coexist in those tissues. However, proviral DNA detection in tissues is not necessarily indicative of a functional latent reservoir. Therefore, total RNA was extracted from frozen tissues that harbored viral DNA and was subjected to 1-step RT-qPCR with HIV-1 *LTR*, *gag*, and *env* primers after DNase I treatment.

**Figure 5:**
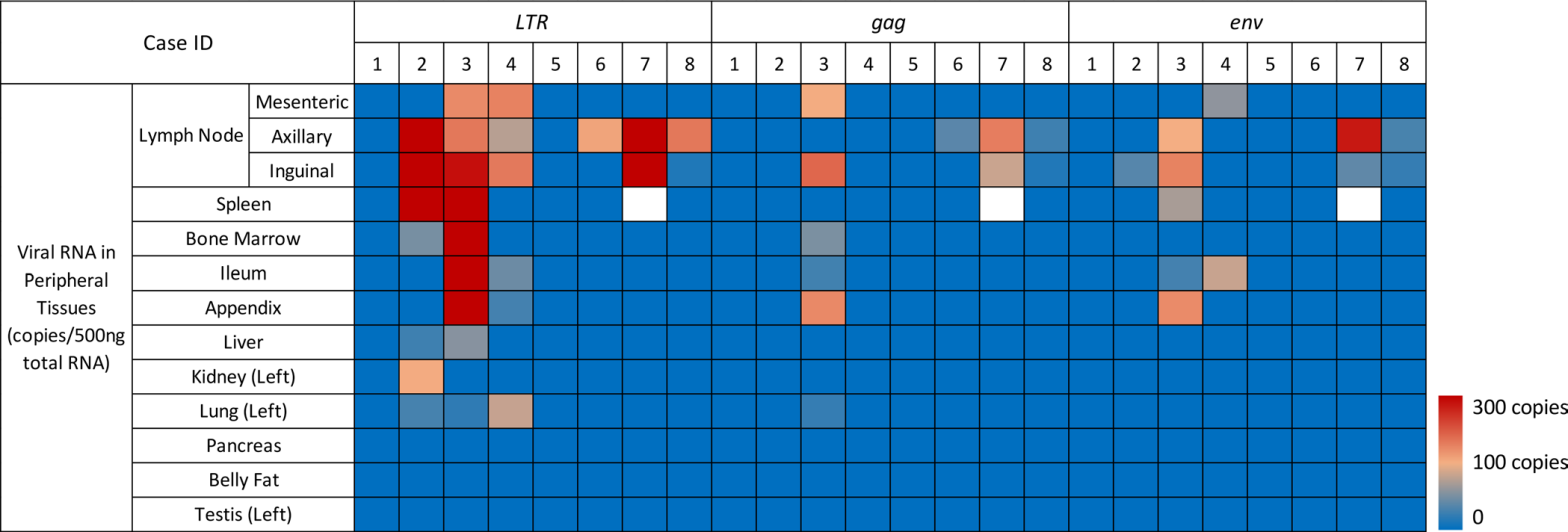
Heatmap of subtype C HIV-1 RNA in aviremic periphery. A heatmap displaying viral RNA transcript (*LTR*, *gag*, and *env*) abundance in the periphery of 8 aviremic subjects was produced by qPCR analysis. Blocks with colors indicate undetectable (0) DNA copy (blue) to increased RNA copies (up to 1,566 copies/500 ng total RNA). HIV-1 RNA copies were identified by qPCR with 500 ng input RNA as template. All samples were analyzed in duplicate qPCR reactions.

**Figure 6:**
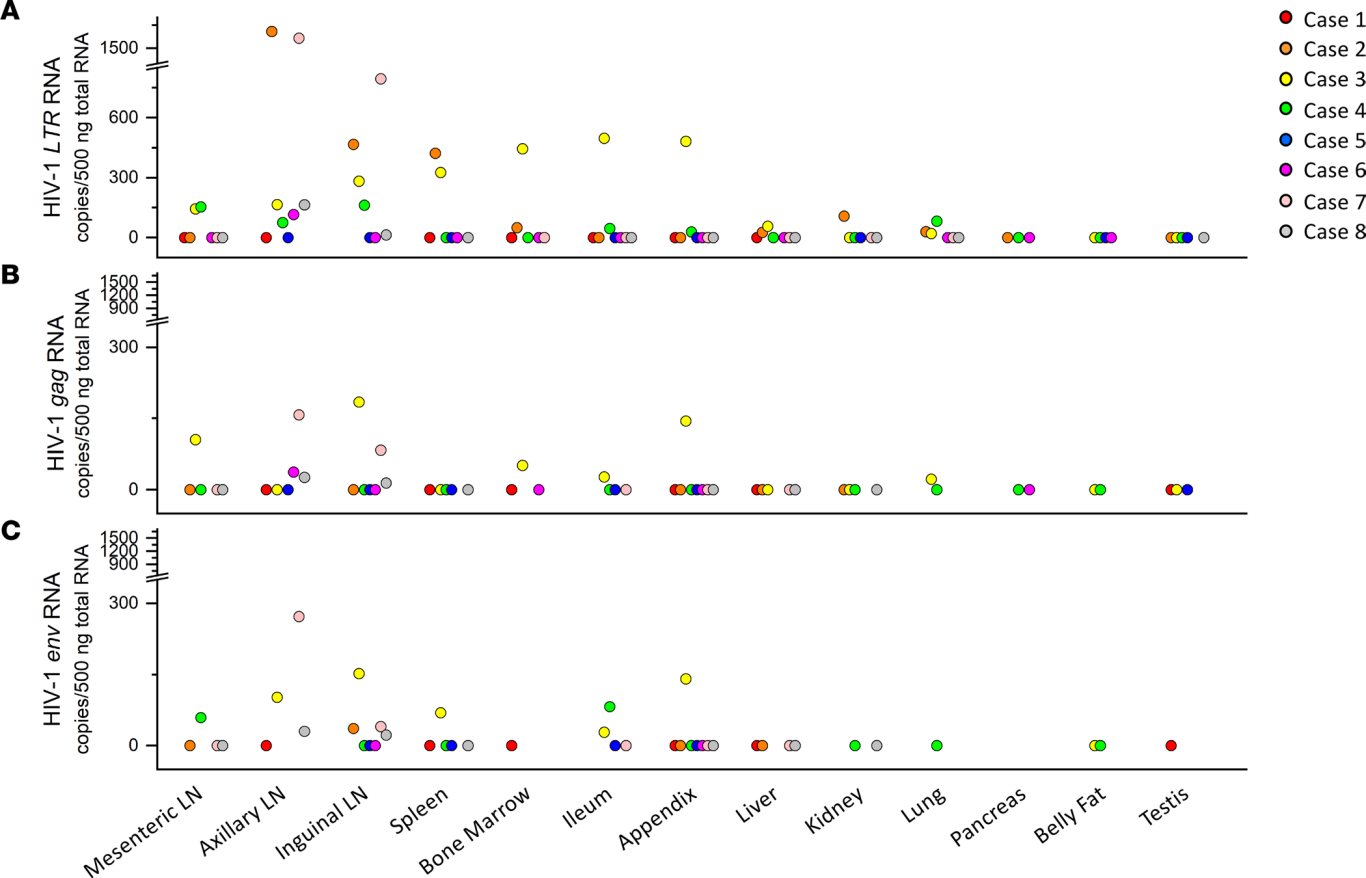
Subtype C HIV-1 RNA quantification in peripheral tissues harboring viral DNA. Scatter plots sorted by different peripheral tissues and colored for different subjects. The *y* axis showed HIV-1 RNA copies/500 ng input RNA. (**A**) HIV-1 *LTR* RNA in the periphery. (**B**) HIV-1 *gag* RNA in the periphery. (**C**) HIV-1 *env* RNA in the periphery. In total, 500 ng input RNA was applied in each qPCR reaction as a template. Viral RNA copy numbers were calculated as the mean of duplicate qPCR reactions.

As seen in Figure 5 and Table 3, HIV-1 *LTR*, *gag*, or *env* RNA was detected in 6 of 8 aviremic cases (undetectable in cases 1 and 5). Subtype C HIV-1 RNA was mainly detected in lymphoid tissues (LNs, spleen, bone marrow), and the predominant site of concordant viral gene expression was the LNs. Among those 6 cases harboring viral RNA in the LNs, 3 cases (cases 6, 7, and 8) did not have any detectable viral RNA besides the LNs. As shown in Figure 6 for case 2 (orange), case 3 (yellow), and case 4 (green) — which had higher overall viral *LTR* DNA burden — viral *LTR* and/or *gag*, and/or *env* RNA, were also sporadically detected in spleen, bone marrow, ileum, appendix, liver, kidney, and lung at variable RNA copies in addition to LNs. The presence of viral RNA indicated potential persistent expression of proviruses in those tissue reservoirs. Pancreas, testis, and belly fat tissues in all aviremic individuals were found to be devoid of detectable subtype C HIV-1 RNA. Additionally, all tissues that expressed viral *gag* or *env* RNA were found to contain viral *LTR* RNA (Figure 6).

In the peripheral tissues harboring HIV-1 DNA, more viral *LTR* RNA (ranging from 0 to 1,609 copies/500 ng total RNA) was detected, compared with viral *gag* RNA (ranging from 0 to 184 copies/500 ng total RNA) (*P* = 0.0045) or *env* RNA (ranging from 0 to 272 copies/500 ng total RNA) (*P* = 0.0049), respectively (Supplemental Figure 7). The main reason is that our *LTR* primers target proviral sequence region encoding leader exon 1, which exists in all HIV-1 transcripts variants. It is also possible that there are undefined promoters outside the provirus, allowing the 3′ end *LTR* gene to transcribe, or some subtype C HIV-1 defective proviruses with deletions in *gag* or *env* region were transcribed, leading to more *LTR* transcripts than *gag* or *env*.

### The appendix as a potentially novel subtype C HIV-1 reservoir.

To our knowledge, the appendix tissue has not previously been investigated or reported as a HIV-1 reservoir. The systematic nature of our sample collection has allowed this first investigation of the appendix as a potential reservoir in fully suppressed subtype C HIV-1 infection. Interestingly, subtype C HIV-1 *LTR*, *gag*, and *env* DNA were detected in the appendix from all aviremic individuals, indicating the very likely presence of full-length subtype C HIV-1 proviruses. HIV-1 *LTR*, *gag*, and *env* RNA were also detected in appendix tissue from case 3, suggesting a possible persistent reservoir in this unique tissue. Blood contamination was unlikely to confound our results from tissue samples because all our cases have undetectable pVL. To identify whether proviral DNA resulted from lymphocyte infiltration into the appendix under inflammation, we first performed histology on appendix FFPE of all aviremic individuals. Classical appendicitis histology would display scattered clusters of dark blue dots (hematoxylin-stained lymphocyte nuclear) in the muscle layer, which we did not observe in any appendix FFPE slide of the 8 aviremic cases, indicating that proviral DNA was not from infiltrated lymphocyte (Supplemental Figure 8). To further determine whether proviral signal quantified in frozen tissues corresponded to parenchymal cell infection and the potential cell types involved, we performed RNAscope with subtype C HIV-1–specific probes on case 3 appendix tissue FFPE.

**Figure 7:**
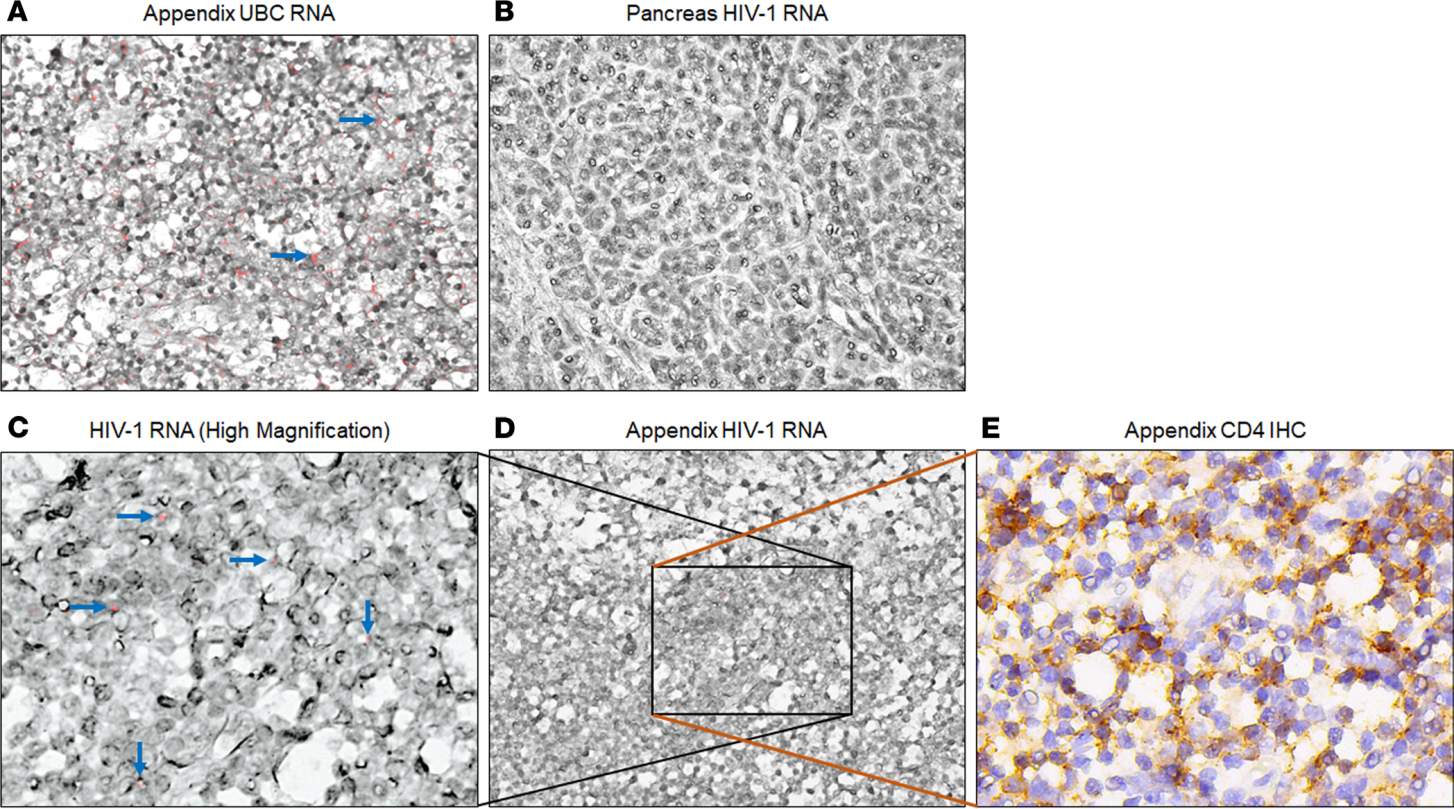
Subtype C HIV-1 DNA and RNA detected in FFPE appendix by RNAscope. Adjacent 6 μm FFPE sections of the appendix of case 3 were stained by RNAscope or IHC. (**A**) *Homo sapiens* ubiquitin C^+^ (UBC^+^) control in the appendix of case 3. (**B**) No HIV-1 DNA or RNA detected in the pancreas (HIV-1 DNA negative by qPCR) of case 3. (**D**) RNAscope for HIV-1 nucleic acid in case 3 appendix. (**C**) Higher magnification of **D** to show HIV-1 DNA and RNA in the appendix of case 3. (**E**) CD4 staining by IHC on the adjacent section of **D**, shown as the same region of **C**. Representative UBC^+^ signals are pointed by blue arrows in **A**, while signals positive for HIV-1 DNA are indicated by horizontal arrows in **C**, shown as pink foci in the nucleus, and HIV-1 RNA signals are pointed by vertical arrows, shown as pink foci outside of the nucleus. CD4 staining is shown brown in **E**. Black rectangles highlight representative regions containing cells positive for HIV-1 RNA and/or DNA and CD4 expression.

Adjacent 6 μm sections were cut from case 3 appendix tissue FFPE and then processed for subtype C HIV-1 DNA and RNA detection by RNAscope and CD4 staining by IHC. Using adjacent sections, we stained different cells in the same area of consecutive slides with CD4 cell markers and a subtype C HIV-1–specific RNAscope probe for HIV-1 nucleic acids. The unique structure of the appendix allowed us to focus precisely on a small area of adjacent slides where the HIV nucleic acid is concentrated. In agreement with our qPCR results, both viral DNA (horizontal arrows) and RNA (vertical arrows) were detected in the case 3 appendix (Figure 7). Viral RNA (pink foci) was localized outside the counterstained nuclei, while viral DNA staining colocalized with the nucleus. The colocalization of viral DNA signal (pink foci) and CD4 staining (brown) in appendix tissue suggested that appendix-associated lymphoid tissues (CD4-expressing cells) can persist as a potentially transcriptionally active and treatment-resistant cellular reservoir for subtype C HIV-1 (Figure 7, C and E). Collectively, the results show that the appendix is a subtype C HIV-1 tissue reservoir in our aviremic individuals analyzed.

## Discussion

Characterizing the distribution and magnitude of HIV-1 reservoirs in tissues other than lymphoid cells, as well as their capacities for reactivation, is a crucial step toward developing optimal strategies to eliminate or permanently silence HIV-1 proviruses in PLWH under ART suppression. The majority of HIV-1 tissue reservoir studies have focused on subtype B HIV-1, which is prevalent in high-resource European and North American countries. However, even in those settings, technical limitations and the difficulty in obtaining appropriate tissues have resulted in only a few HIV-1 studies that encompassed diverse human tissues systems, such as CNS tissues, lymphoid tissues, or GI tract tissues, to investigate HIV-1 subtype-B tissue reservoirs (34, 44). Instead, blood samples or those collected through routine biopsy methods, such as colonoscopy or surgically obtained colon tissue, have been the focus (38, 51). Alternatively, macaques or humanized mice have been systematically sampled (17, 20, 52). These approaches have indeed provided a substantial body of work about subtype B HIV-1 reservoirs. Despite the global prevalence of subtype C HIV-1 and its detection in PBMCs of ART naive individuals (45), the localization, magnitude, and expression activity of those reservoirs after ART suppression is relatively unknown. In part, this is because pathology resources and individuals with appropriate skills and access to instrumentation for systematic postmortem investigations are uncommon in sub-Saharan Africa, which is the epicenter of subtype C HIV-1 infection. This study focused on sensitive recruitment of defined postmortem tissue collection, systematic sampling, and careful fixation and processing focused toward unambiguous downstream molecular applications on subtype C HIV-1–infected postmortem tissues, through our long-term collaborative pathology laboratory resource at the University of Zambia Teaching Hospital (UTH) in Lusaka, Zambia. This unique postmortem collection of frozen and FFPE specimens allowed us to accurately identify potential anatomical locations of subtype C HIV-1 reservoirs.

In previous studies, the brain has been reported as a reservoir of subtype B HIV-1 even under ART suppression, despite being somewhat immunologically privileged and separated from the circulating immune system by the BBB (53–55). It is generally believed that the relatively poor penetration of ART drugs into the brain due to the protection of the BBB, and therefore the limited capacity of ART to eliminate infiltrated HIV-1–infected cells or cell-free HIV-1 virions, is the main reason that the brain serves as subtype B HIV-1 reservoir.

Currently, little is known about the extent to which the brain serves as a reservoir for subtype C HIV-1, and likewise, long-term neurocognitive impacts of subtype C HIV-1 are unclear in the ART-treated and virally suppressed individuals. A previous study showed that subtype B HIV-1 DNA was detected in 48 of 87 (55.2%) brain tissue samples from HIV-1–infected ART-suppressed patients at levels > 200 HIV-1 DNA copies/1 × 10^6^ cell equivalents (44). In contrast, our laboratory had detected subtype C HIV-1 proviral DNA at low and variable levels in CNS tissues, regardless of ART success or failure (46). However, the small size of the ART-suppressed cohort (*n* = 4) and lack of systematic collection of other major organ tissues had limited the exploration of subtype C HIV-1 reservoirs throughout the body. In this study, we demonstrate that HIV-1 DNA was only detected in 12 of 64 (18.8%) of Zambian brain tissue samples from subtype C infected but aviremic individuals. And when detectable, the brain tissue proviral levels were quite low (<100 HIV-1 DNA copies/1 × 10^6^ cell equivalents). Our findings are consistent with previous studies that have suggested lower neurotropism of subtype C in comparison with subtype B HIV-1. First, clinically, subtype C HIV-1–infected patients showed a reduced incidence of HAND (56). In addition, mice with severe combined immunodeficiency (SCID) injected with subtype C HIV-1–infected macrophages exhibited less cognitive deficits and brain pathology than those infected with subtype B HIV-1 (57). Furthermore, subtype C HIV-1 was found to have slower replication kinetics in monocyte-derived macrophages, resulting in lower levels of macrophage-mediated neurotoxicity in vitro (58). These investigations suggest subtype C HIV-1 differentially accesses the CNS parenchyma in comparison with subtype B, indicating that the brain is an unfavorable or inaccessible reservoir site for subtype C HIV-1. However, despite our sampling of 8 distinct brain regions, it is still possible that subtype C proviral DNA preferentially integrates and establishes reservoirs in very specific tissue locations in the CNS that we failed to sample. In addition, the presence of detectable proviral sequences despite the absence of viral RNA in the brain suggests the possibility that some latent subtype C HIV-1 reservoirs in the CNS may be intact in some PLWH. Studies on subtype B HIV-1 have shown that microglia, astrocytes, and macrophages can be infected by HIV-1 (59–61). Unfortunately, these approaches cannot be readily applied to the brain sections from aviremic cases to determine the subtype C HIV-1–infected cell types in the brain, because the subtype C HIV-1 DNA copy was so low in the brain that the total number of cells needed in order to detect infected cells with detectable viral DNA is beyond what can be observed in a single brain section.

Infection and reservoir formation in peripheral tissues has been adequately investigated for subtype B HIV-1. Lymphoid and GI tract tissues had been identified as primary sites of subtype B HIV-1 infection and persistence (34). In agreement with these observations, we also detected subtype C HIV-1 reservoirs in lymphoid and GI tract tissues. These sites (LNs, spleen, ileum, and appendix) showed significantly higher levels of subtype C HIV-1 DNA than other peripheral tissues. Consistent with subtype B studies (62, 63), we likewise found subtype C HIV-1 RNA primarily in lymphoid and GI tract tissues. We detected subtype C HIV-1 DNA in diverse peripheral tissues, including testis and adipose tissues, which remain the subject of some debate as subtype B HIV-1 reservoirs. Nevertheless, subtype C HIV-1 proviruses were also detected in all other peripheral tissues analyzed but with variable levels, and they differ among individuals. Our results suggest that, even under ART suppression, subtype C HIV-1 tissue reservoirs are unevenly distributed and occur at various magnitudes throughout the body, posing a huge challenge for a functional or sterile cure of subtype C HIV-1.

The coexistence of 3 viral genes indicated the potential for full-length or nearly full–length HIV-1 proviruses in those tissues. Single genome amplification and sequencing (SGA/S) would increase the sensitivity of integrity analysis of proviral genome, but these methods require high cellular input, which may limit the ability to explore subtype C HIV-1 reservoirs. In tissues with relatively low infected cell numbers — for instance, the CNS, adipose tissue, and testis — these analyses would be prohibited. In such cases, our 3-gene amplification method provides a viable approach to assess the potential intactness of subtype C reservoirs. However, recent studies utilizing intact proviral DNA analysis (IPDA) have shown that both intact and defective subtype B HIV-1 proviruses exist in tissue reservoirs, with the majority being defective (64–66). In agreement with those findings, we also detected lack of stoichiometric equality of *LTR*, *gag*, and *env* DNA detection in the same tissue, suggesting that intact and defective subtype C HIV-1 proviruses might also coexist. Notably, among 94 tissues of our cohort harboring subtype C HIV-1 DNA, 48 tissues (51.1%) have detectable *LTR* but lack *gag* or *env* DNA, indicating that the majority of proviruses are defective in subtype C HIV-1 reservoirs.

It would be important to determine whether proviruses in the reservoirs are replication competent, whether they are intact, and whether they express viral RNA and protein to better determine whether they may stay latent or persistent expressing, particularly in those infected individuals under ART suppression. Although quantitative viral outgrowth assay (QVOA) is considered as a gold standard assay for detecting and measuring potential for reactivation of replication-competent HIV-1 virus (67–70). Our limitation is that QVOA has not yet been successfully conducted from postmortem tissues. From tissues harboring viral DNA, we tested for expression of viral *LTR*, *gag*, and *env* and found that subtype C HIV-1 RNA can only be detected in peripheral (non-CNS) tissues, suggesting that proviruses in those tissue reservoirs might be replication competent and might persistently express viral RNA. We attempted IHC for HIV-1 p24 CA on the FFPE tissue the inguinal LN from case 3, which had the highest *gag* RNA copies (184 copies per 500 ng RNA input) in the corresponding frozen tissue, but there was no detectable viral p24 in the FFPE sections (Supplemental Figure 9). It is possible there was very low–level anti-p24 protein expressed in FFPE tissues, below the detection sensitivity of IHC or the efficiency of anti-p24 antibody we used.

Notably, the appendix, a GI tract tissue rich in lymphoid cells and recently described as having important immune functions (71), has not previously been reported as an HIV-1 tissue reservoir. In our analysis, we detected viral *LTR*, *gag*, and *env* DNA in all 8 appendix tissues, implying the presence of at least some intact subtype C genomes in the appendix. Moreover, viral RNA was also been detected in 2 appendix tissues (cases 3 and 4). Interestingly, previous studies had revealed that the prevalence of appendicitis in PLWH is 4-fold higher than that in uninfected individuals (72–74), and opportunistic infections and immune reconstitution inflammatory syndrome (IRIS) at the initiation stage of ART were thought to be the reasons. Given our findings, there may also be active HIV-1 proviruses existing in the appendix of those other cases studied, and persistent HIV-1 expression could be another reason for the higher occurrence of appendicitis in HIV-infected people. However, the colocalization of CD4 staining with proviral DNA signal is not sufficient to identify the infected cell type in the appendix because CD4 is expressed by multiple immune cell types, such as T cells, macrophages, and DCs. The definition of the specific cell type of the appendix HIV-1 reservoir needs to be further studied via multiple immunostaining with different cell markers. Additionally, whether the appendix can harbor ART-resistant HIV-1 also needs to be determined.

The study had some clear limitations. First, we only had 8 aviremic cases for full-body postmortem evaluation. A bigger cohort size could provide more information on subtype C HIV-1 reservoirs. Second, as a result of deaths in the community, as opposed to being under clinical care, our study individuals lacked detailed clinical information, including HIV diagnosis date, ART initiation date, ART adherence, HIV load data, additional health status, and CD4 cell counts prior to death. This information is often difficult to obtain even in studies with subtype B under different settings; thus, it has been very challenging to gather such information in Zambia, where clinical records are not typically computerized or linked. Furthermore, subtype C HIV-1 is responsible for approximately 99% of HIV infections in Zambia, and we failed to sample a single subtype B HIV-1–infected Zambian individual in the past 4 years of our study. It is also not possible to obtain autopsy samples from HIV/AIDS diseased cases in North America or Europe or from countries with subtype B infection to establish tissue-matched controls for subtype C HIV-1, especially due to the need to systematically collect 8 brain and 13 peripheral subtype B HIV-1–infected biopsy or autopsy tissues from a single individual. Additionally, although dPCR results from several tissues supported our qPCR results, the sensitivity of qPCR may limit our ability to capture extremely low levels of subtype C HIV-1 proviruses in some tissues. Besides case 3 appendix FFPE tissues, RNAscope was also performed on case 2 mesenteric LN, case 3 axillary LN, and case 4 appendix tissues, estimated to have lower copies of proviral DNA by qPCR. Nevertheless, no proviruses were detectable in those 3 FFPE tissues by RNAscope, as shown in Supplemental Figure 10. It is possible that low copy number of the HIV-1 proviruses may not be evenly distributed and was not in the embedded section of the tissues we investigated; furthermore, RNAscope may not be sensitive enough to identify very low levels of proviruses in subtype C HIV-1–infected aviremic FFPE tissues. Finally, since all the individuals were deceased, the generalizability of our findings to healthy PLWH cannot be ascertained.

In summary, this study demonstrates that subtype C HIV-1 DNA copies varied among virally suppressed individuals and anatomical compartments, and the CNS is poorly accessed or not a robust reservoir. To our knowledge, this is the first study to our knowledge that systematically quantifies the magnitude and distribution of subtype C HIV-1 reservoirs in human tissues from different compartments throughout the body of subtype C HIV-1–infected individuals under ART suppression. Our findings that the appendix is a potentially important persistent reservoir but that CNS is not for subtype C HIV-1 is substantial. Whether subtype B or other HIV-1 subtypes can establish a persistent reservoir in the appendix needs to be examined. Whether other non–subtype B HIV-1 is similar to subtype C HIV-1 that do not efficiently establish persistent infection in the CNS also needs to be determined. Considering that over 50% prevalence of subtype C HIV-1 among all HIV-1 infections and the relatively high economic burden caused by life-long ART, especially in low-source regions, our findings indicate that only few and very low–level subtype C HIV-1 DNA with no detectable viral RNA in the brain — but readily detectable in the appendix — could provide useful information for the cure effort against subtype C HIV-1, including optimization and administration of ART toward the various tissue reservoirs involved.

## Methods

### Study setting, ethics statement, and sample collection.

Zambia is a sub-Saharan African country where HIV-1 prevalence remains high and infection with subtype C HIV-1 is predominant (75, 76). UTH is the largest hospital in Zambia, and its pathology department performs approximately 1,300 forensic autopsies annually, 17% of which are on HIV-1^+^ individuals. In order to obtain samples, when a potential autopsy case arrived at the UTH morgue, a clinical officer or study nurse counselor approached the family members of the deceased to determine whether the deceased met study inclusion criteria with regard to time of death, and to seek informed consent for the postmortem collection of tissue specimens. Because sociodemographic and lifestyle behaviors could not be directly assessed from the deceased, the nurse counselor, working with the clinical officer, surveyed the next of kin — the individual who provided consent for tissue collection — for demographic information about the deceased. The nurse counselor utilized a validated WHO-ASSIST survey instrument designed to collect social demographic and behavioral data. At the same time, we also accessed clinical records to extract portions of the patient’s medical history, including ART regimen and duration. Only study personnel had access to the study database, and only deidentified data was made available for data analysis. Postmortem samples were obtained using standardized procedures. Within 48 hours of the individual’s death, multiple CNS and collateral tissues were collected and stored by 2 methods: (a) half of the sample was stored at –80°C and (b) the other half was fixed at 4% paraformaldehyde and then further processed for FFPE tissue blocks. In addition, plasma samples from all individuals were obtained and stored at –80°C until use.

### HIV-1 serology and qPCR for plasma HIV-1 viral load quantification.

HIV-1 serological test was conducted using HIV Rapid Test or Alere Determine HIV-1/2 Ag/Ab Combo test in Zambia (77), and those serological results were further verified in the laboratory at the University of Nebraska-Lincoln with HIV-1-2.0 First Response kit (Premier Medical Corporation Limited, Daman, India). To quantify the plasma HIV-1 viral loads, total RNA in a plasma sample was extracted in 1 batch with standard AcroMetrix HIV-1 setting (5 × 10^2^/mL, 5 × 10^3^/mL, 5 × 10^4^/mL, 5 × 10^5^/mL, or 5 × 10^6^/mL) (Thermo Fisher Scientific) via QIAamp Viral RNA Kit (Qiagen) with DNase I treatment to remove any DNA. Same volume (5 μL) plasma samples and AcroMetrix HIV-1 setting were subjected to qPCR as triplicates using the RNA UltraSense One-Step qPCR System (Invitrogen) to detect the pVLs. Primers and probes against HIV-1 *LTR* were used. The procedure for qPCR was 50°C for 15 minutes, 95°C for 2 minutes to synthesize cDNA, and 40 cycles by 95°C for 15 seconds for DNA denaturation and 60°C for 1 minute for annealing and elongation. The cutoff of plasma HIV-1 *LTR* RNA detection was 70 copies/mL.

### Extraction of genomic DNA and RNA from postmortem tissues.

Small pieces of snap-frozen tissues were collected in 2 sets of 1.5 mL centrifuge tubes by 1 mm biopsy punch tool (Robbins Instruments) on dry ice for DNA and RNA extraction, respectively. For genomic DNA extraction, 600 μL cell lysis buffer and 10 μL proteinase K solution (Qiagen) were added in tubes containing the tissues; then, the samples were incubated overnight in a 55°C water bath. Each sample was then incubated at 37°C with 10 μL RNase-A solution (Qiagen) for 1 hour to remove any RNA. After RNA removal, the samples were chilled on ice for 3 minutes before adding 200 μL protein precipitation solution into each tube; the samples were then vigorous vortexed and spun down at 16,000*g* for 10 minutes at 4°C to precipitate protein. During centrifugation, 400 μL isopropanol and 0.5 μL glycogen was added into a new tube. The supernatant was transferred to the new tube containing isopropanol and glycogen for genomic DNA precipitation by inverting tubes 50 times, followed by centrifugation at 16,000*g* for 1 minute at room temperature. The supernatant was then discarded, and the DNA pellet at the bottom of the tube was washed once with 70% ethanol and air dried for 5–10 minutes. Then, 30–100 μL hydration solution (Qiagen) was added in the tube to resuspend the DNA pellet. After a brief vortex, the tubes were incubated at 65°C for at least 1 hour to completely dissolve the DNA pellet. The genomic DNA samples were further purified by phenol chloroform, and the DNA concentrations were measured via Qubit dsDNA BR assay kit (Invitrogen). The DNA samples were stored at –20°C until use.

For total RNA extraction from tissues, 700 μL QIAzol lysis reagent (Qiagen) was added to each tube containing the fresh tissue. After 5 minutes of incubation of the homogenate, 140 μL chloroform followed by centrifugation at 12,000*g* at 4°C for 15 minutes to separate phases. Total RNA from the top aqueous phase was then extracted with Qiagen miRNeasy mini kit (Qiagen) according to manufacturer’s protocol. On-column DNase I treatment was also applied during the RNA extraction. The RNA concentrations were measured via Qubit RNA BR assay kit (Invitrogen); the RNA samples were then stored at –80°C for the subsequent viral RNA analyses.

### HIV-1 subtyping.

To confirm HIV-1 clade C infection in the aviremic cases studied, nested PCR with primers against gp120 V3 loop was performed with the following procedure for both rounds: polymerase activation at 95°C for 10 minutes, 35 cycles of denaturation at 95°C for 30 seconds, annealing at 55°C for 30 seconds and elongation at 72°C for 2 minutes, then 1 cycle of 72°C for 7 minutes. In first round, 100 ng genomic DNA was used as template with *envelope* primers (Forward: 5’-CCTGCTGGTTATGCGATTCTAAA-3’; Reverse 1: 5’-ACCTCCTGCCACATGTTTATAATTTG-3’) were applied. Then, 2 μL of the first-round PCR product was utilized as a template for the second round with another pair of *envelope* primers (Forward: 5’-CCTGCTGGTTATGCGATTCTAAA-3’; Reserve 2: 5’-CAATAGAAAAATTCTCCTCTACAATTAAA-3’). Products from the second round–nested PCR were checked for size and purified via gel extraction; they were then sequenced with the forward primer. RIP- and BLAST-based HIV-1 genotyping tool were used for subtyping sequence alignments.

### qPCR for HIV-1 DNA quantification.

In total, 100 ng extracted genomic DNA from each tissue was used as template for both quality control GAPDH and HIV-1 *LTR*, *gag*, and *env* DNA detection. TaqMan Universal PCR Master Mix (Thermo Fisher Scientific) was used with 300 nM each of HIV-1 *LTR* U5 forward and reserve primers (5’-GCCTCAATAAAGCTTGCCTTGA-3’; 5’-GGGCGCCACTGCTAGAGA-3’) and Fam-labeled HIV-1 *LTR* probe (/56-FAM/5’-CCAGAGTCACACAACAGACGGGCACA-3’/3BHQ_1). For HIV-1 *gag*, each HIV-1 *gag* forward and reserve primers (5’-CAAGCAGCCATGCAAATGTT-3’; 5’-ATGTCACTTCCCCTTGGTTCTC-3’) and Fam-labeled HIV-1 *gag* probe (/56-FAM/5’-CCTGGTGCAATAGGCCCTGC-3’/3BHQ_1/) were used. For HIV-1 *env*, each HIV-1 *env* forward and reserve primers (5’-TGTTCCTTGGGTTCTTGGGAG-3’; 5’-TGCTGCTGCACTATACCAGAC-3’) and HEX-labeled HIV-1 *env* probe (5’-HEX-TCTGGCCTGTACCGTCAGCG-IFBQ-3’) were used. Genomic 8E5 cellular DNA containing a single proviral HIV-1 genome in each cell was mixed with uninfected PBMC genomic DNA to generate standards that contain 5, 10, 100, and 1000 8E5 HIV-1 copies in 100 ng total genomic DNA. A DNA sample from a uninfected individual was used as negative control. Standards and tissue DNA samples were applied in QuantStudio 3 Real-Time PCR System (Thermo Fisher Scientific) in triplicates with a reaction volume of 20 μL for each. The thermal cycling condition utilized in qPCR were: 95°C for 15 seconds for polymerase activation and then 40 cycles of 95°C for 15 seconds for DNA denaturation and 60°C for 1 minute for annealing and elongation.

### dPCR for HIV-1 DNA quantification.

HIV-1 DNA copies in the genomic DNA from LN, appendix, testis, and occipital lobes of cases 2, 3, and 4 were also determined by dPCR performed on QIAcuity One instrument with QIAcuity probe PCR kit (Qiagen). Briefly, genomic DNA samples were first digested with restriction enzyme SalI, whose recognition site does not exist in the target HIV-1 *LTR* sequences to reduce sample viscosity and increase template accessibility. Human β-globin internal control and HIV-1 *LTR* DNA in triplicates with 10 ng digested DNA β-globin detection and 700 ng digested DNA as templates were used. Each dPCR reaction mixture in total volume of 40 μL consisted of 1× QIAcuity probe master mix, 800 nM each of HIV-1 *LTR* primers, 400 nM FAM-labeled HIV-1 *LTR* probe, template DNA, and molecular grade water. The reaction mixtures were then transferred to a 26,000 partitions per well 24-well nanoplate (Qiagen) and sealed with a plate seal; they were then amplified in the QIAcuity One thermocycler. The thermal cycling condition used for dPCR was: 95°C for 2 minutes, 40 cycles of 95°C for 15 seconds for DNA denaturation, and 60°C for 30 seconds for annealing and elongation. The threshold for dPCR positivity was determined using signals for viral DNA detection in digested genomic DNA from the corresponding tissues of an HIV-1^–^ individual, and DNA from the mesenteric LN of a HIV-1 viremic case was used as positive control (Supplemental Table 5). By dividing the mixture into partitions, the instrument measured and calculated the target sequence copies after end-point PCR cycling based on the presence or absence of a fluorescent signal for human β-globin or HIV-1 *LTR* DNA in every individual partition. Analysis was performed using the software suite (QIAcuity Software Suite), providing the concentration in copies per mL of *LTR* sequence; this was also accomplished for human β-globin.

### qPCR for HIV-1 tissue RNA load quantification.

To quantify HIV-1 RNA copies in tissues that harbor viral DNA, total RNA in the tissues was extracted as described above. In total, 500 ng total RNA and 2.5 μL AcroMetrix HIV-1 standards (5; 50; 500; 5,000; and 50,000 copies of cell free HIV-1) were subjected into qPCR in triplicates by RNA UltraSense One-Step RT-qPCR System (Invitrogen) to detect the tissue viral RNA loads. The primers and probes against the HIV-1 *LTR*, *gag*, and *env* region used for DNA copy determination were also applied for viral RNA quantification. The thermocycle procedure for qPCR was the same as that for pVL determination.

### RNAscope in situ hybridization (ISH) and IHC.

To identify the tissue parenchymal location of HIV-1 nucleic acids and infected cell type, 6 μm of adjacent sections was cut from FFPE appendix tissues of individual case 3 and then subjected for RNAscope ISH and IHC staining. For viral DNA and RNA detection, one section of each appendix tissue was stained with DNAscope antisense probe using RNAscope 2.5 HD red reagent kit (Advanced Cell Diagnostics) based on the RNAscope 2.5 HD red reagent kit instruction and a previously reported protocol (78). Other adjacent sections were stained with *Homo sapiens* ubiquitin C (UBC) probe as positive control and dihydrodipicolinate reductase (dapB) probe as negative control by RNAscope. One of adjacent slides was stained for CD4 cell marker by IHC with mouse anti–human CD4 monoclonal antibody (M7310, 1:100, Dako), and the staining signal was developed with substrate DAB by Dako Envision and Peroxidase kit. Matched IgG 1 isotype controls were applied in IHC staining to confirm signal specificity. For viral p24 protein detection, IHC was performed on FFPE inguinal LN from case 3, monoclonal mouse anti–HIV p24 antibody was used (M0857, 1:20, Dako). The stained slides were then digital scanned by MoticEasyScan Pro instrument (Motic) according to the instruction manual provided by the manufacturer.

### Statistics.

To examine the differences in HIV-1 *LTR* DNA copies between brain and peripherial tissues of the ART-suppressed individuals, and the differences in HIV-1 *LTR* DNA copies between group 1 (LNs, spleen, and appendix) and group 2 (other peripheral tissues) of the ART-suppressed individuals, both Wilcoxon matched-pairs signed-rank test and nonparametric Mann-Whitney *U* test were used to assess the differences between comparison groups. GraphPad Prism 9 (GraphPad Software) was utilized for statistical analyses. All tests were 2-tailed and *P* values < 0.05 were considered as significant.

### Study approval.

This study excluded individuals younger than 18 years and collected informed consent from the family members of the deceased. All aspects of this study were reviewed and approved by the University of Zambia Biomedical Research Ethics Committee (UNZABREC) in keeping with the Zambian national research policies governing the research use of postmortem samples.

## Author contributions

Conceptualization was contributed by CW. Data curation was contributed by ZL and GK. Formal analysis was contributed by ZL and JTW. Funding acquisition was contributed by CW. Investigation was contributed by ZL and CW. Methodology was contributed by ZL, GK, and PJ. Project administration was contributed by CW. Resources were contributed by PJ and CW. Supervision was contributed by CW. Visualization was contributed by ZL, GK, and JTW. Writing of the original draft was contributed by ZL. Review and editing and the manuscript were contributed by ZL, JTW, and CW.

## Supplementary Material

Supplemental data

## Figures and Tables

**Table 3:**
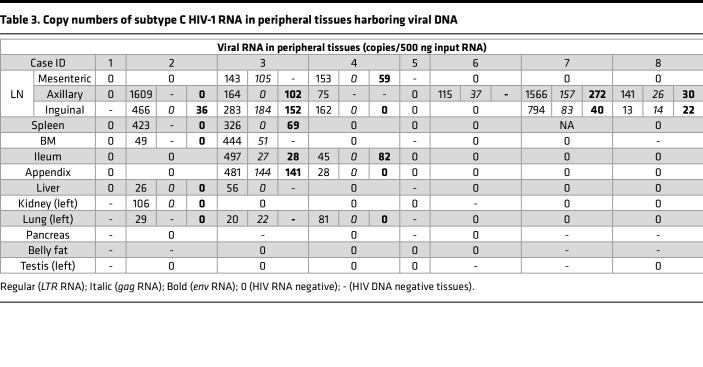
Copy numbers of subtype C HIV-1 RNA in peripheral tissues harboring viral DNA

**Table 1:**
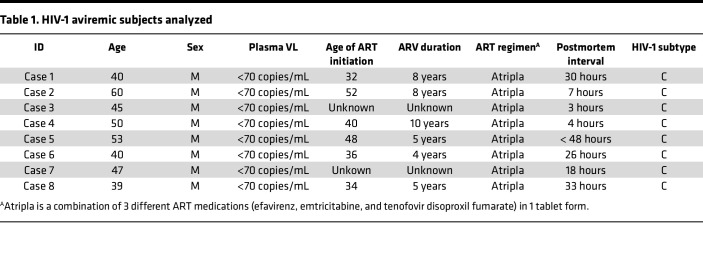
HIV-1 aviremic subjects analyzed

**Table 2:**
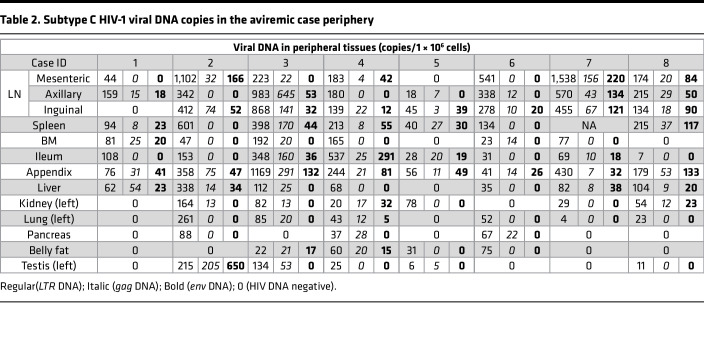
Subtype C HIV-1 viral DNA copies in the aviremic case periphery

## Version 1 10/24/2022

Electronic publication
