# Supplementary material for: Subtype C HIV-1 reservoirs throughout the body in ART-suppressed individuals
Source: JCI Insight. 2022 Oct 24;7(20):e162604. doi: 10.1172/jci.insight.162604 (PMC9714794; doi:10.1172/jci.insight.162604)
Supplement: Supplemental data [file jciinsight-7-162604-s172.pdf]

Supplementary Materials

Supplementary Table 1. **HIV-1 subtyping results of 8 aviremic subjects.**

Case ID	Tissues for Sequencing	Subtype Results
1	Axillary Lymph Node	C
2	Testis	C
3	Appendix	C
4	Mesenteric Lymph Node	C
5	Inguinal Lymph Node	C
6	Inguinal Lymph Node	C
7	Mesenteric Lymph Node	C
8	Appendix	C

Supplementary Table 2. **Similar amplification efficiencies of different HIV-1 DNA primers.**

8E5 DNA	HuBG		LTR		gag		env	
	Ct Mean	Ct SD	Ct Mean	Ct SD	Ct Mean	Ct SD	Ct Mean	Ct SD
0.05ng (7copies)	34.770	0.350	35.573	0.691	36.295	0.908	35.566	1.183
0.1ng (15copies)	33.253	0.224	34.081	0.308	34.511	0.454	34.592	0.685
1ng (150copies)	29.968	0.101	30.584	0.009	31.134	0.170	30.675	0.076
10ng (1500copies)	26.478	0.093	27.159	0.050	27.542	0.024	27.163	0.045

The amplification efficiencies of HIV-1 *LTR*, *gag*, and *env* primers had been tested by qPCR. Gradient 8E5 genomic DNA were used as templates. The mean of Ct value and Ct standard deviations of triplicate qPCR reactions are shown. Primer against human beta-globin (HuBG) gene was also included in the efficiency test. Similar amplification efficiencies of different HIV-1 DNA primers were observed.

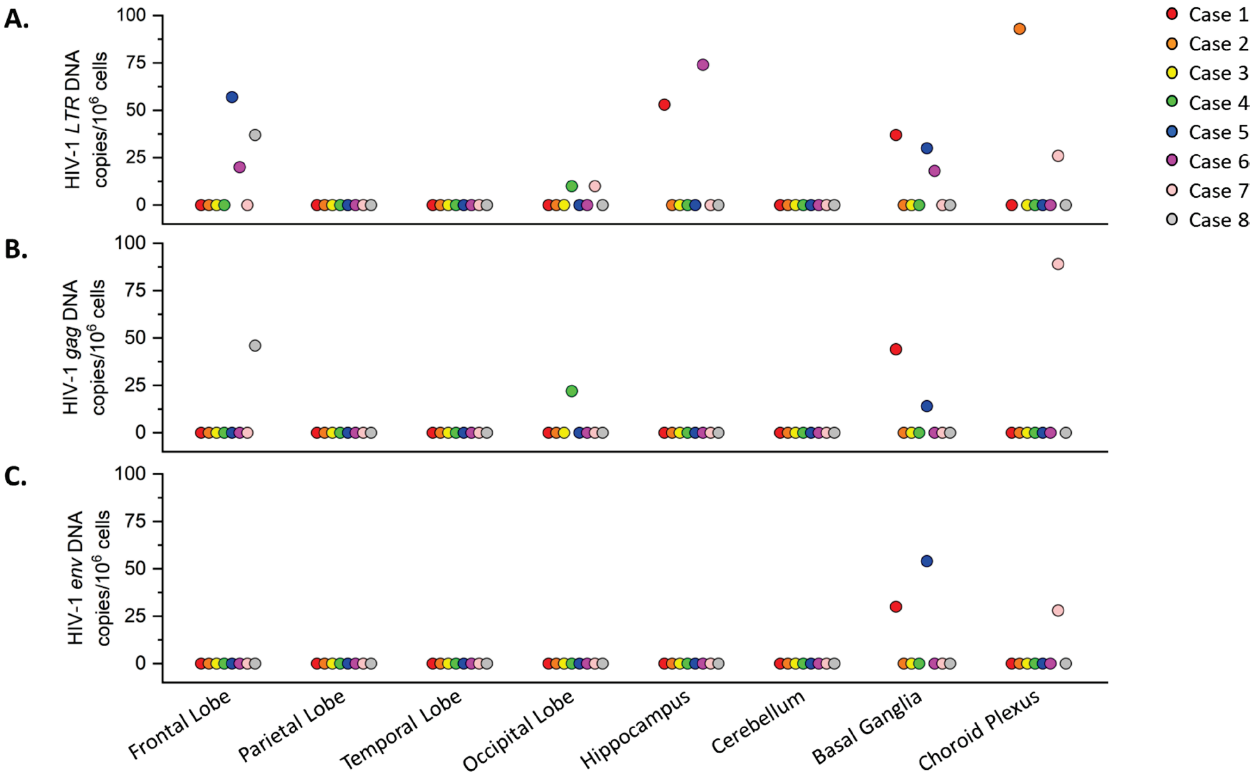

Supplementary Figure 1. Subtype C HIV-1 DNA quantification in different brain regions. Scatter dot plots by different brain regions and by different subjects colored. The y axis showed HIV-1 DNA copies/10^6 cells. (A) HIV-1 *LTR* DNA in the brain. (B) HIV-1 *gag* DNA in the brain. (C) HIV-1 *env* DNA in the brain. HIV-1 DNA copies were identified by qPCR with 100 ng genomic DNA as template. Viral DNA copy numbers were calculated as the mean of triplicate qPCR reactions and normalized to 1 million cells. The dots represent the mean of triplicate qPCR reactions for the HIV-1 DNA copies.

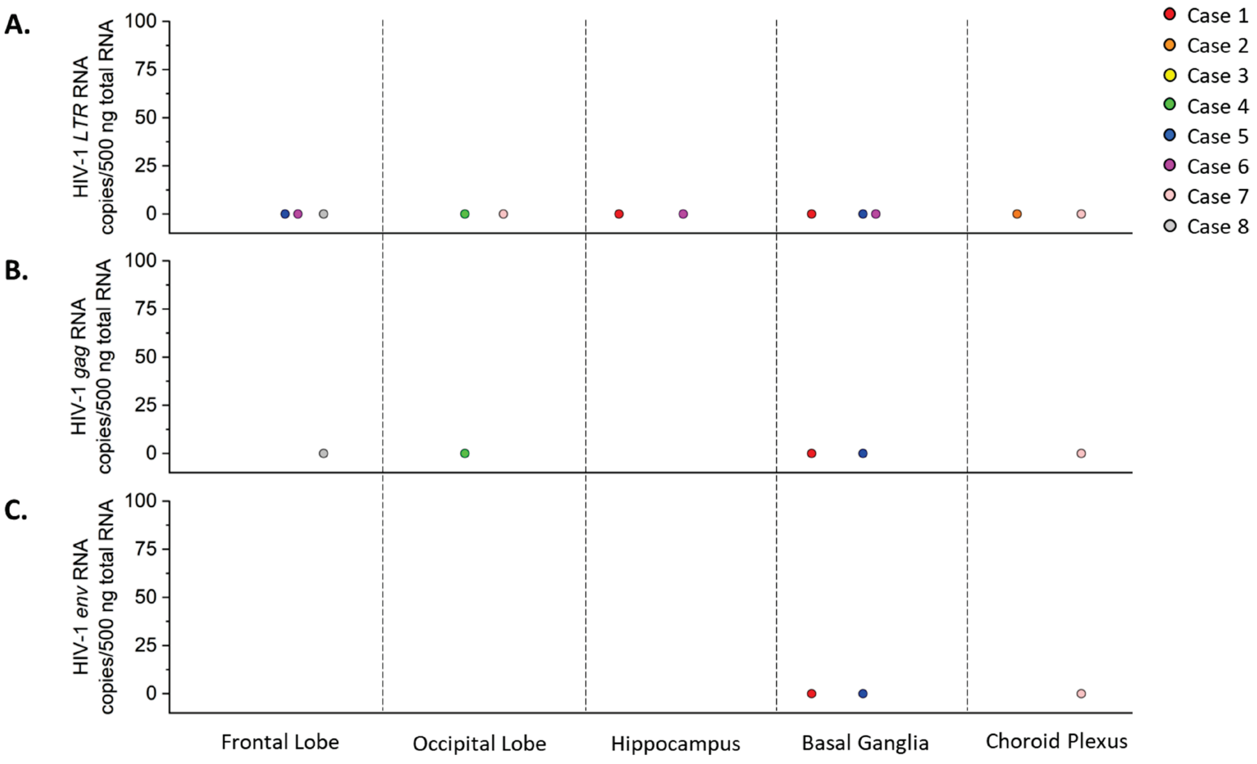

Supplementary Figure 2. **Subtype C HIV-1 RNA was not detectable in brain.** Scatter dot plots sorted by 5 different brain regions harboring viral DNA and by different subjects colored. The y axis showed HIV-1 RNA copies/500 ng total RNA. (A) HIV-1 *LTR* RNA in the brain. (B) HIV-1 *gag* RNA in the brain. (C) HIV-1 *env* RNA in the brain. HIV-1 RNA copies were identified by one-step reverse transcription (RT)-qPCR with 500 ng total RNA input as template. Viral RNA copy numbers were calculated as the mean of duplicate RT-qPCR reactions.

Supplementary Table 3. **Differences in the subtype C HIV-1 reservoir size between the brain and periphery.**

A. Difference of viral DNA copies

Column B	Peripheral
vs.	vs.
Column A	Brain
Mann Whitney test	
P value	<0.0001
Exact or approximate P value?	Exact
P value summary	****
Significantly different (P < 0.05)?	Yes
One- or two-tailed P value?	Two-tailed
Sum of ranks in column A,B	45 , 126
Mann-Whitney U	0
Difference between medians	
Median of column A	8.375, n=9
Median of column B	123.5, n=9
Difference: Actual	115.1
Difference: Hodges-Lehmann	115.1

B. Difference of number of tissues

Table Analyzed	Brain vs Periphery		
P value and statistical significance			
Test	Chi-square		
Chi-square, df	59.43, 1		
z	7.709		
P value	<0.0001		
P value summary	****		
One- or two-sided	Two-sided		
Statistically significant (P < 0.05)?	Yes		
Effect size	Value	95% CI	
Relative Risk	0.2355	0.1379 to 0.3811	
Reciprocal of relative risk	4.246	2.624 to 7.253	
Attributable risk (P1 - P2)	0.6086	0.4998 to 0.7613	
NNT (reciprocal of attrib. risk)	1.643	1.314 to 2.001	
Odds ratio	0.05910	0.02811 to 0.1317	
Reciprocal of odds ratio	16.92	7.595 to 35.57	
Sensitivity	0.1277	0.07456 to 0.2100	
Specificity	0.2877	0.1965 to 0.4001	
Positive Predictive Value	0.1875	0.1106 to 0.2997	
Negative Predictive Value	0.2039	0.1374 to 0.2917	
Likelihood Ratio	0.1792		
Methods used to compute CIs			
Relative Risk	Koopman asymptotic score		
Attributable risk (P1 - P2)	Newcombe/Wilson with CC		
Odds ratio	Baptista-Pike		
Sensitivity, specificity, etc.	Wilson-Brown		
Data analyzed	HIV-1 DNA+	HIV-1 DNA-	Total
Brain	12	52	64
Periphery	82	21	103
Total	94	73	167
Percentage of row total	HIV-1 DNA+	HIV-1 DNA-	
Brain	18.75%	81.25%	
Periphery	79.61%	20.39%	

(A) Mann Whitney test showed there was a significant difference between brain and periphery for HIV-1 DNA copies since the p value < 0.0001. (B) This frequency of viral DNA harboring tissues in periphery (79.6%) is significantly higher than that in the brain (18.8%) (P<0.0001).

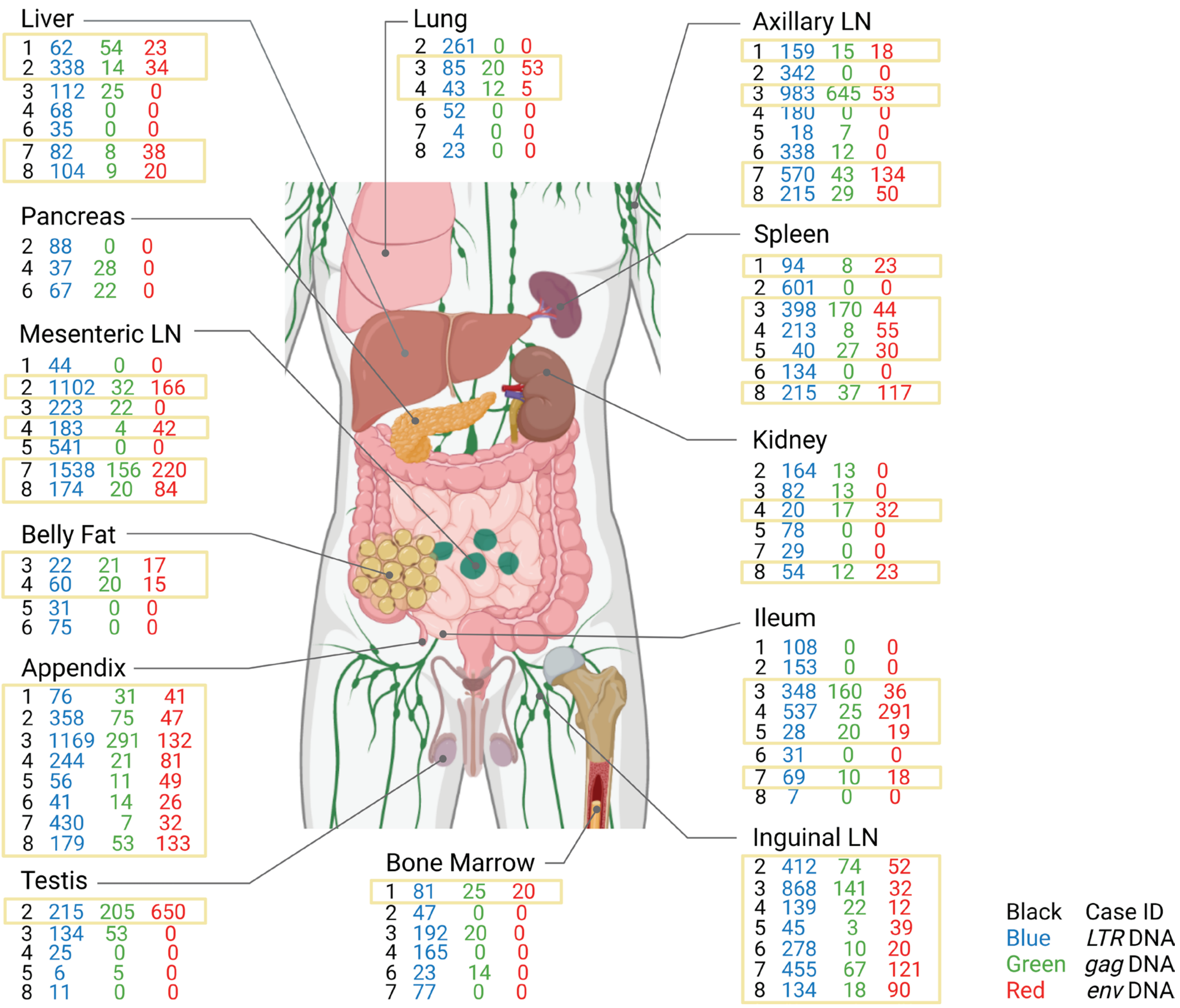

Supplementary Figure 3. **Subtype C HIV-1 viral DNA copies in the aviremic case periphery.** The copy numbers of viral *LTR*, *gag*, and *env* DNA in the periphery of 8 aviremic subjects were summarized. HIV-1 *LTR* (blue), *gag* (green), and *env* (red) DNA copy numbers were calculated as the mean of triplicate qPCR reactions and represent DNA copies/10^6 cells. The rectangles mark peripheral tissues harboring all three viral DNA, indicating potential intact viral genome in those tissues.

Supplementary Table 4. **Difference of HIV-1 DNA copies between group 1 (LNs, spleen, ileum, and appendix) and group 2 (the other peripheral tissues).**

A. Wilcoxon Test

Column B	Other Peripheral Tissues
vs.	vs.
Column A	LN_Spleen_Ileum_Appendix
Wilcoxon matched-pairs signed rank test	
P value	0.0039
Exact or approximate P value?	Exact
P value summary	**
Significantly different (P < 0.05)?	Yes
One- or two-tailed P value?	Two-tailed
Sum of positive, negative ranks	0.000 , -45.00
Sum of signed ranks (W)	-45.00
Number of pairs	9
Number of ties (ignored)	0
Median of differences	
Median	-149.0
How effective was the pairing?	
rs (Spearman)	0.6500
P value (one tailed)	0.0333
P value summary	*
Was the pairing significantly effective?	Yes

B. Mann Whitney Test

Column B	Other Peripheral Tissues
vs.	vs.
Column A	LN_Spleen_Ileum_Appendix
Mann Whitney test	
P value	0.0019
Exact or approximate P value?	Exact
P value summary	**
Significantly different (P < 0.05)?	Yes
One- or two-tailed P value?	Two-tailed
Sum of ranks in column A,B	119 , 52
Mann-Whitney U	7
Difference between medians	
Median of column A	180.8, n=9
Median of column B	34.38, n=9
Difference: Actual	-146.4
Difference: Hodges-Lehmann	-146.4

(A) Wilcoxon matched-pairs signed rank test showed there was a significant difference between two groups for HIV-1 DNA copies, p value < 0.01. (B) Mann Whitney test also showed there was a significant different between two groups for HIV-1 DNA copies, p value < 0.01.

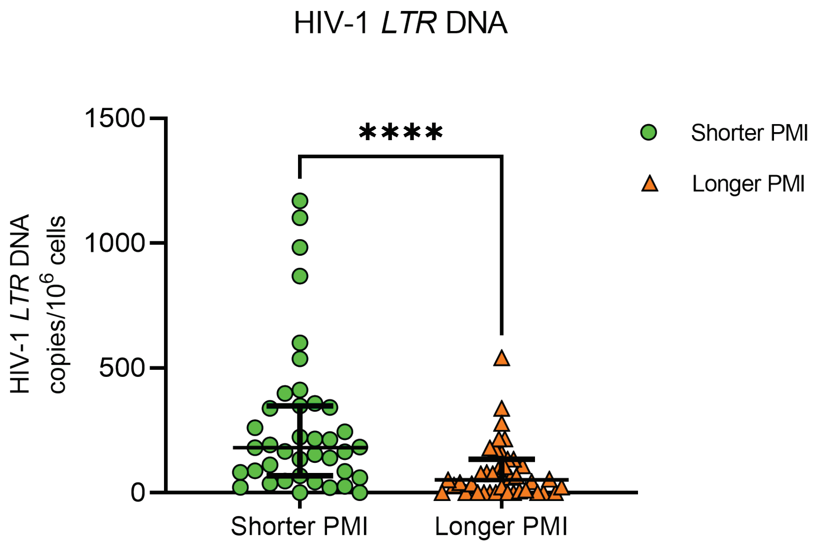

Supplementary Figure 4. **Cases with shorter PMI harbored higher overall viral DNA in the periphery than cases with longer PMI.** Three cases with relative shorter postmortem interval (PMI), cases 2 (7 hours), 3 (3 hours) and 4 (4 hours) had relatively higher overall viral *LTR* DNA burden in the periphery than that other three cases with longer PMI, cases 1 (30 hours), 6 (26 hours), and 8 (33 hours) ($P < 0.0001$). Total 39 peripheral tissues in 3 cases with shorter PMI were marked as green dots and 39 peripheral tissues in cases with longer PMI were indicated as brown triangles.

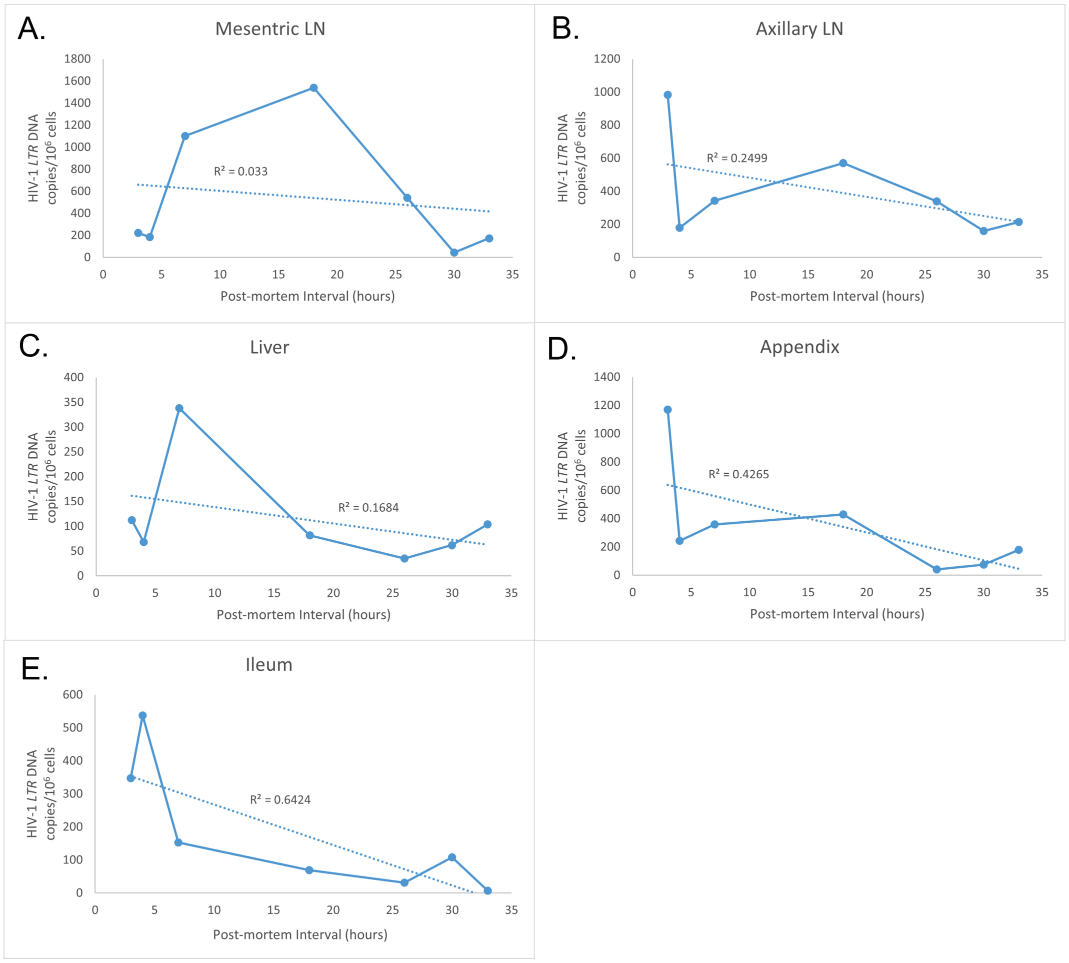

Supplementary Figure 5. **PMI is not correlated to the level of viral DNA detected in different peripheral tissues.** The correlation of post-mortem interval (PMI) with the level of proviral DNA detected in different peripheral tissues were tested. (A) There is no correlation of PMI and viral DNA level in mesenteric LN (correlation coefficient = -0.1817, R^2 = 0.033). (B) There is no correlation of PMI and viral DNA level in axillary LN (correlation coefficient = -0.4999, R^2 = 0.2499). (C) There is no correlation of PMI and viral DNA level in the liver (correlation coefficient = -0.4103, R^2 = 0.1684). (D) There is no correlation of PMI and viral DNA level in the appendix (correlation coefficient = -0.6531, R^2 = 0.4265). (E) There is an inverse correlation of PMI and viral DNA level in the ileum (correlation coefficient = -0.8015, R^2 = 0.6424).

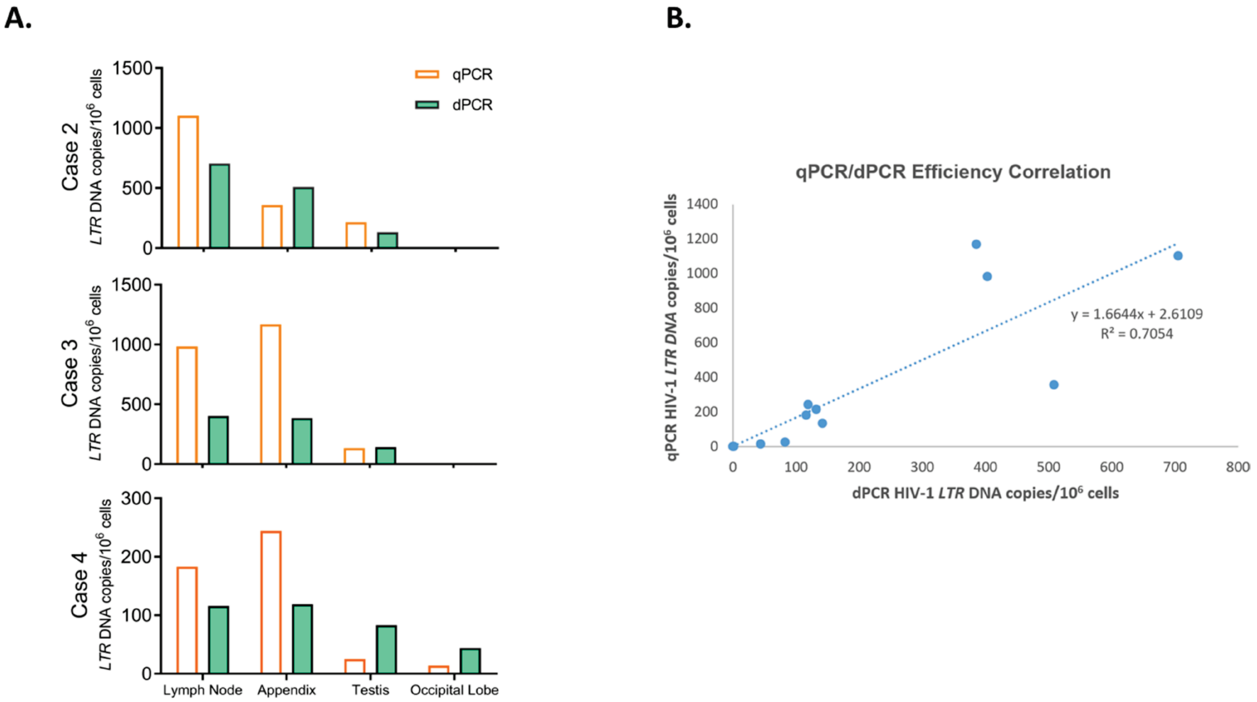

Supplementary Figure 6. **Digital PCR (dPCR) results support qPCR results.** (A) The histograms showed the absolute viral *LTR* DNA copies determined by dPCR (green shaded bar) and the corresponding viral *LTR* DNA copies identified by qPCR (open bar) in LN, appendix, testis and occipital lobe from cases 2, 3 and 4. (B) the correlation efficiency between qPCR and dPCR based on the detected HIV-1 *LTR* DNA copies. Absolute HIV-1 *LTR* DNA copy numbers were calculated as the mean of triplicate dPCR reactions and normalized to 1 million cells using the human beta globin as the internal reference. 700 ng genomic DNA was used as template in each dPCR reaction. The dots represent the mean of the HIV-1 DNA copies and R^2 value for the slope is 0.7054.

Supplementary Table 5. **HIV-1 *LTR* DNA quantification by digital PCR (dPCR) in different tissues.**

	Tissues	dPCR (Backgound Removed)	qPCR (HIV-1 LTR)
HIV- Control	Mesenteric LN (LNM)	**0** copies/10^6 cells	
	Appendix	**0** copies/10^6 cells	
	Spleen	**0** copies/10^6 cells	
	Lung	**0** copies/10^6 cells	
	Cerebellum	**0** copies/10^6 cells	
	Occipital Lobe	**0** copies/10^6 cells	
HIV+ Control	Mesenteric LN (LNM)	**10660** copies/10^6 cells	
Case 2	Mesenteric LN (LNM)	**705** copies/10^6 cells	**1102** copies/10^6 cells
	Appendix	**509** copies/10^6 cells	**358** copies/10^6 cells
	Lung	**900** copies/106 cells	**261** copies/10^6 cells
	Testis	**132** copies/106 cells	**215** copies/10^6 cells
	Occipital Lobe	**0** copies/10^6 cells	**0** copies/10^6 cells
Case 3	Axillary LN (LNA)	**403** copies/10^6 cells	**983** copies/10^6 cells
	Appendix	**386** copies/10^6 cells	**1169** copies/10^6 cells
	Lung	**324** copies/106 cells	**85** copies/10^6 cells
	Testis	**142** copies/106 cells	**134** copies/10^6 cells
	Occipital Lobe	**2** copies/10^6 cells	**0** copies/10^6 cells
Case 4	Mesenteric LN (LNM)	**116** copies/10^6 cells	**183** copies/10^6 cells
	Appendix	**119** copies/10^6 cells	**244** copies/10^6 cells
	Lung	**176** copies/106 cells	**43** copies/10^6 cells
	Testis	**83** copies/106 cells	**25** copies/10^6 cells
	Occipital Lobe	**44** copies/10^6 cells	**14** copies/10^6 cells

Genomic DNA from corresponding tissues of a non-infected individual was used as negative control, and from the mesenteric LN of a viremic case was used as positive control. Background signals were removed based on the threshold.

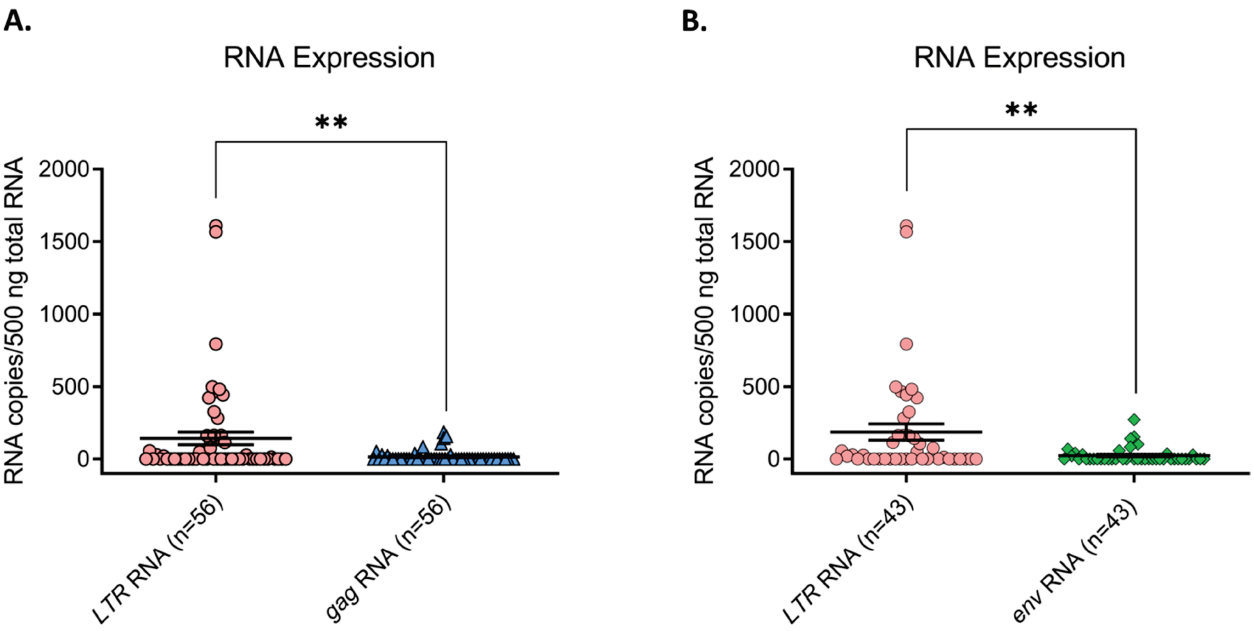

Supplementary Figure 7. **More detectable *LTR* RNA than *gag* or *env* RNA in viral DNA harboring peripheral tissues.** (A) There was a significant higher HIV-1 *LTR* RNA burden than *gag* RNA in peripheral tissues harboring both viral *LTR* and *gag* DNA (n=56), p value < 0.01. (B) There was a significantly higher HIV-1 *LTR* RNA burden than *env* RNA in peripheral tissues harboring both viral *LTR* and *env* DNA (n=43), p value < 0.01.

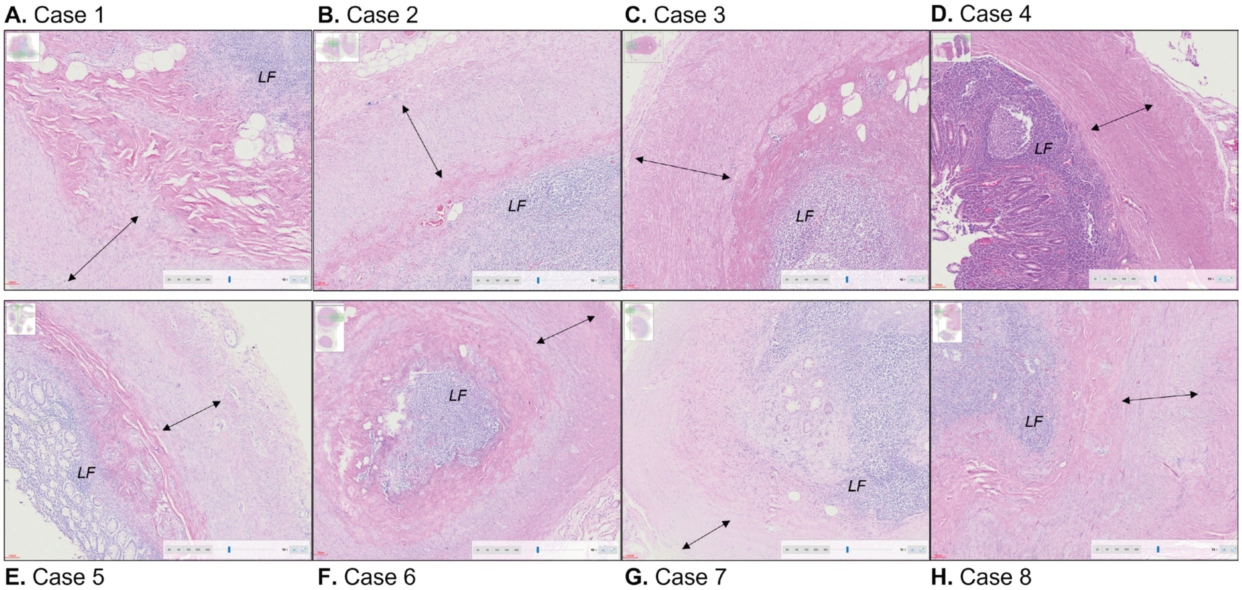

Supplementary Figure 8. **No detectable inflammation in the appendix of the aviremic subjects.** The inflammatory status, as a pathological indicator of appendicitis of the 8 aviremic appendix tissues were analyzed by histology. Classical appendicitis histology would display scattered clusters of dark blue dots (hematoxylin-stained lymphocyte nuclear) in the muscularis propria, which was not observed in the appendix FFPE slides of 8 aviremic cases (panel A-H). Black arrows mark the muscularis propria and LF means lymphoid follicle. Thumbnails and magnification of representative areas (10X) are also shown.

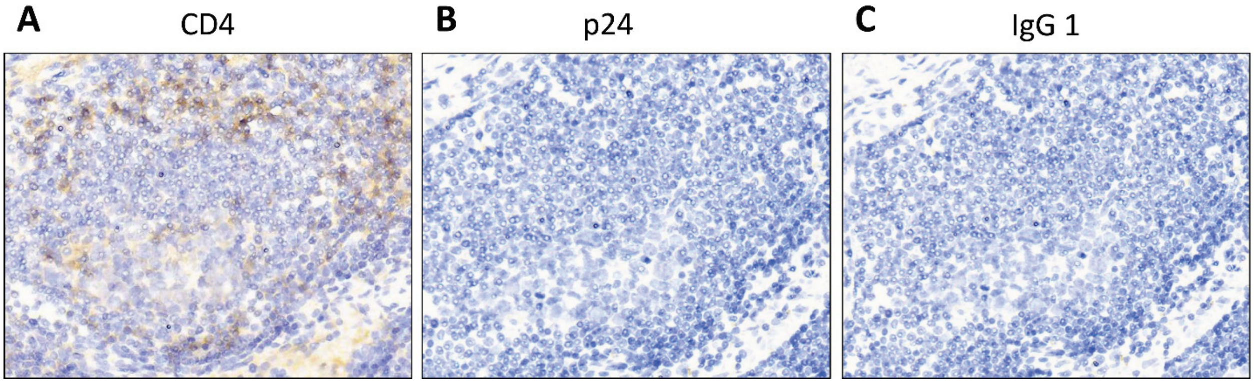

Supplementary Figure 9. **No detectable HIV-1 p24 protein in the inguinal LN FFPE of case 3.** IHC was performed on the case 3 inguinal LN tissue FFPE, which contains highest p24 encoding *gag* RNA copies, with different antibodies against (A) cell surface marker CD4, (B) viral protein p24, and (C) isotype control IgG 1. Nuclei are shown as blue color stained by hematoxylin. CD4 expression are shown in brown color.

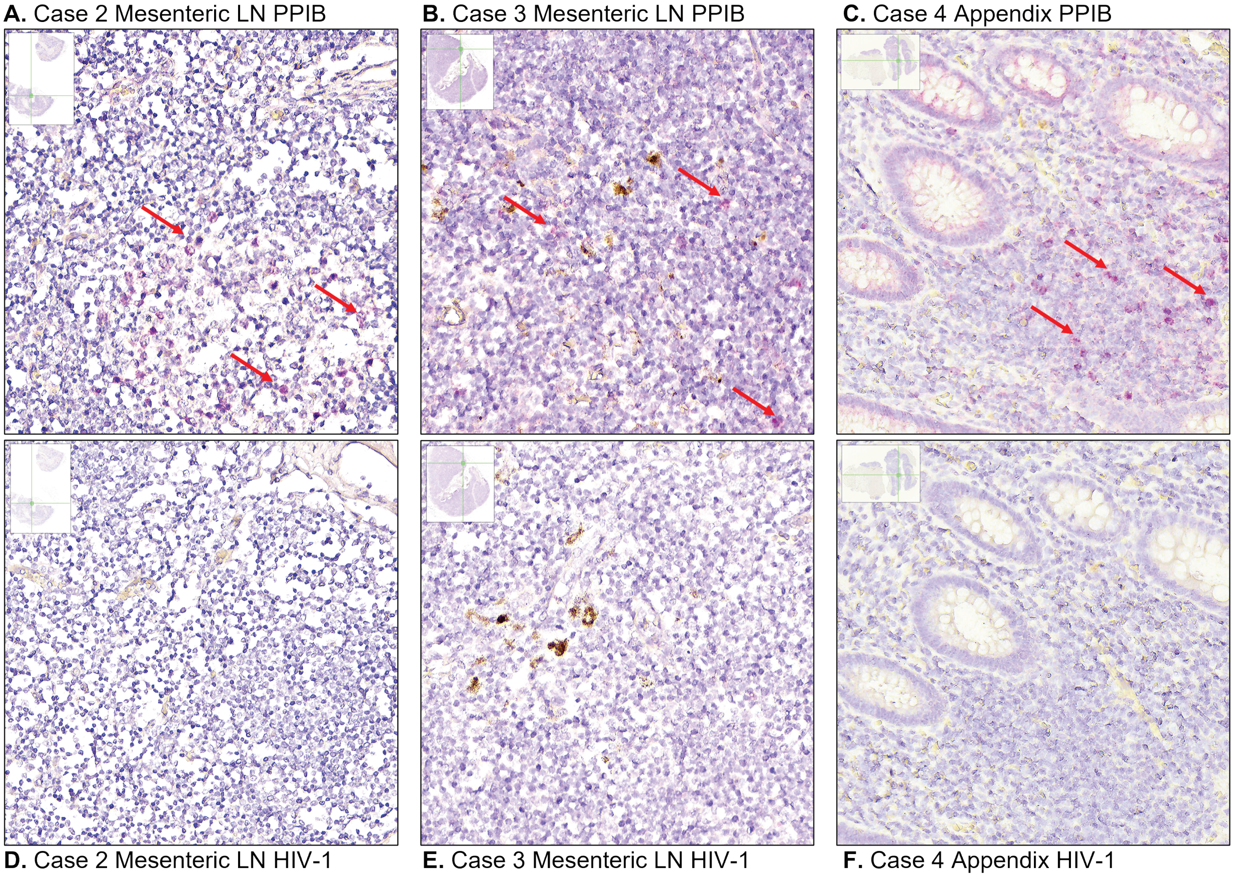

Supplementary Figure 10. No detectable subtype C HIV-1 provirus in case 2 mesenteric LN, case 3 axillary LN, and case 4 appendix by RNAscope. (A-C) RNAscope on case 2 FFPE mesenteric LN, case 3 FFPE axillary LN, and case 4 FFPE appendix showed that cellular RNA *PPIB*, as positive control for RNAscope system, can be detected in those 3 tissues. Red arrows indicate representative *PPIB* RNA signals (pink foci). (D-F) Subtype C HIV-1 provirus was not detected in those 3 tissues by RNAscope.
